# Standards of Evidence for Efficacy, Effectiveness, and Scale-up Research in Prevention Science: Next Generation

**DOI:** 10.1007/s11121-015-0555-x

**Published:** 2015-04-07

**Authors:** Denise C. Gottfredson, Thomas D. Cook, Frances E. M. Gardner, Deborah Gorman-Smith, George W. Howe, Irwin N. Sandler, Kathryn M. Zafft

**Affiliations:** 1 University of Maryland, College Park, MD USA; 2 Northwestern University, Evanston, IL USA; 3 University of Oxford, Oxford, UK; 4 University of Chicago, Chicago, IL USA; 5 George Washington University, Washington, DC USA; 6 Arizona State University, Tempe, AZ USA

**Keywords:** Standards of evidence, Policy

## Abstract

A decade ago, the Society of Prevention Research (SPR) endorsed a set of standards for evidence related to research on prevention interventions. These standards (Flay et al., Prevention Science 6:151–175, 2005) were intended in part to increase consistency in reviews of prevention research that often generated disparate lists of effective interventions due to the application of different standards for what was considered to be necessary to demonstrate effectiveness. In 2013, SPR’s Board of Directors decided that the field has progressed sufficiently to warrant a review and, if necessary, publication of “the next generation” of standards of evidence. The Board convened a committee to review and update the standards. This article reports on the results of this committee’s deliberations, summarizing changes made to the earlier standards and explaining the rationale for each change. The SPR Board of Directors endorses “The Standards of Evidence for Efficacy, Effectiveness, and Scale-up Research in Prevention Science: Next Generation.”

## Introduction

A decade ago, the Society of Prevention Research (SPR) endorsed a set of standards for evidence related to research on prevention interventions. These standards were intended in part to increase consistency in reviews of prevention research that often generated disparate lists of effective interventions due to the application of different standards for what was considered to be necessary to demonstrate effectiveness. A committee of prevention scientists, chaired by Brian Flay, was convened to determine the requisite criteria that must be met for preventive interventions to be judged “tested and efficacious” or “tested and effective.” The resulting standards were articulated in a document published in *Prevention Science* (Flay et al. 2005) and summarized in more succinct form on the Society’s web page (http://www.preventionresearch.org/StandardsofEvidencebook.pdf). This work is frequently cited not only to justify methods used in studies but also in debates about what standards should be applied in prevention research. It has been influential in the policy world as well.

In 2013, SPR’s Board of Directors decided that the field has progressed sufficiently to warrant a review and, if necessary, publication of “the next generation” of standards of evidence. It noted that important research has been conducted in the past decade that might alter design recommendations for efficacy studies, that the earlier standards did not provide sufficient guidance on standards for replication, and that the field had made important strides in understanding prerequisites for scaling up of effective interventions that should now be incorporated into the SPR standards. Hence, the Board convened a second committee, chaired by Denise Gottfredson, to review and update the standards. This article reports on the results of this committee’s deliberations, summarizing changes made to the earlier standards and explaining the rationale for each change.

In interpreting the SPR Board’s charge, the Committee had to first clarify the intended purposes for the revised document. Standards can be defined to map into current practices to help maintain them or they can be purposefully set higher to encourage growth in the field. We opted for the latter. For example, we identified numerous ways in which early trials of an intervention can help to provide information (e.g., about costs and variability in the quality of implementation) that may not be critical in the early stages of development but that will become critical later. We also added a standard to encourage testing of the mediating pathways that are specified in the theory of the intervention during the efficacy trial phase. Clearly, it will take many years before researchers begin to build these elements into their research proposals and before funding agencies adapt their priorities to encourage inclusion of these elements in funded research. We nevertheless believe it is important to articulate standards that will provide direction to the field as it addresses key scientific questions to better understand the effects of preventive interventions and their potential public health impact.

Given the forward-looking intent for the document, it is important to clearly state that we would regard it as premature and inappropriate for many of the standards contained in this document to be translated into requirements for interventions to be recognized as effective for prevention practice today. Many organizations have developed standards to guide prevention practice (e.g., European Monitoring Centre for Drugs and Drug Addiction 2011; U.S. Preventive Services Task Force, http://www.uspreventiveservicestaskforce.org/) or have applied reasoned standards to identify effective interventions based on research currently available (e.g., University of Colorado’s Blueprints for Healthy Youth Development (http://www.blueprintsprograms.com/); Center for Disease Control and Prevention’s Guide to Community Preventive Services (http://www.cdc.gov/epo/communityguide.htm); Coalition for Evidence-Based Policy Top-Tier Evidence Initiative (http://toptierevidence.org/); U.S. Department of Education’s What Works Clearinghouse (http://ies.ed.gov/ncee/wwc/); Office of Justice Programs’ CrimeSolutions.gov; and SAMHSA’s National Registry of Evidence-Based Programs and Practices (NREPP; http://nrepp.samhsa.gov/). The standards articulated in this document are intended to elevate the rigor of the scientific evidence that will be available in the future as these efforts are renewed and revised. Likewise, although we anticipate that the field will evolve as these standards begin to guide publication and funding decisions in the future, it would be inappropriate to use the standards articulated in this document as a checklist of items that must be present in a single work.

### Definitions and Organization

The charge of the earlier committee was to “determine the most appropriate criteria *for prevention programs and policies* to be judged *efficacious, effective, or ready for dissemination*” (Flay et al. 2005, p. 152, italics added). We have opted to use the broader term, intervention, throughout this document to include programs, policies, and practices aimed at improving health and well-being or at reducing disease and related problems. These interventions can target or affect units ranging from subperson systems (e.g., the immune system) to defined populations of individuals, to entire communities, states, or nations. They include diverse activities ranging from changes in individuals’ diets to influence brain chemistry, to sessions for parents to improve family management practices, to efforts to alter school climate and culture, to media messages, to community infrastructure changes such as improving the water supply, paving roads, or installing lights in parking lots, to legislative action. An “evidence-based intervention” (EBI) is an intervention that has been tested in research meeting the efficacy standards described below and that has been demonstrated in this research to achieve statistically and practically meaningful improvements in health and wellness or reductions in disease or related problems.

An assumption on which these standards are based is that high-quality research on such interventions will lead to beneficial change. We do not assume that such research is the *only* mechanism for producing beneficial change or even the most effective mechanism. The recent highly effective tobacco control movement, for example, involved communication of the risks of smoking, advocacy, policy making, litigation, as well as making smoking cessation supports widely available to smokers. Sound research on the risks of smoking and the effectiveness of specific strategies for reducing smoking were necessary building blocks for this movement to occur, but not sufficient to produce change without a range of additional efforts aimed at translating the research into broad change. As important as these nonresearch activities are producing change, they are not the main focus of this document. Here we articulate standards for high-quality research on specific interventions that will hopefully provide the basis for sound decision-making about which interventions should be promoted.

The earlier standards were organized according to efficacy research, effectiveness research, and broad dissemination efforts. Flay et al. (2005) defined efficacy trials as studies of programs or policies delivered under optimal conditions, and effectiveness trials as studies conducted in real-world conditions. They noted that these types of trials differ in the amount of researcher control over potentially confounding factors, with efficacy trials most often involving high researcher control over implementation and effectiveness trials involving less. Flay et al. (2005) stated that effectiveness trials require an additional burden of proof above and beyond that required for efficacy trials because, for example, it is necessary to demonstrate that the intervention was in fact delivered under real-world conditions and that the outcomes are generalizable to the population targeted by the intervention. Further, Flay et al. (2005) argued that even interventions that have been demonstrated to be effective in the real world might not be ready for broad dissemination. They therefore provided additional criteria that must be met in order to justify a decision to broadly disseminate.

Flay et al. (2005) treated the “broad dissemination” category differently than the efficacy and effectiveness categories. They did not consider standards for *research* on broad dissemination efforts, but rather assumed that once an intervention had cleared all hurdles related to efficacy and effectiveness, further rigorous research on its effects was not necessary as long as fidelity to the proven model was demonstrated. The earlier standards instead recommended that monitoring and evaluation tools suitable for use by practitioners be made available to adopting organizations so that they could demonstrate to their constituencies that their prevention dollars were well spent. The Flay et al. (2005) document was therefore organized around the traditional preventive intervention research cycle (Mrazek and Haggerty 1994), which depicted a mostly linear progression (although feedback across stages was anticipated) for prevention research beginning with basic research on the nature of the problem to be addressed, progressing through the development of interventions, pilot testing, efficacy trials, effectiveness trial, and finally leading to broad dissemination. Each phase focused on testing a different set of questions.

This framework was appropriate to describe the prevention field 10 years ago, when Prevention Science was dominated by studies of risk and protective factors and evaluations of newly developed interventions. In the past 10 years, as it became clear that preventive interventions do indeed produce desired outcomes at least under optimal conditions, the emphasis in Prevention Science has shifted more toward understanding how these EBIs can be implemented on a broader scale to produce larger impacts on entire populations. A recent report from an SPR task force on type 2 translational research (Spoth et al. 2013) addresses the challenges to translating EBIs into broader usage and sets an agenda for needed research to advance this new science of translation. This task force recognizes that successful translation of EBIs into broader usage will require a more elaborated and less linear progression of research than is implied by the progression from efficacy to effectiveness to dissemination in the prior SPR standards. Specifically, it suggests that research on factors that are likely to influence the success of scale-up efforts should be incorporated throughout each phase of the development and testing of EBIs. It also recommends a more collaborative, practice-oriented framework for developing EBIs than is implied in the traditional preventive intervention research cycle. Such a collaborative approach, implemented across all stages of the development of EBIs, could yield rich information early on about the factors that are likely to influence the success of attempts to move the EBI into broader usage later. Spoth et al. (2013) also call for an increase in research on the outcomes of scale-up efforts. This research is necessary because neither prevention interventions nor alternatives to these interventions are static. Interventions may become more or less effective relative to the usual state that would be present in the absence of the EBI. Only by studying outcomes of scale-up efforts will we learn how interventions work in these new circumstances.

We incorporated this updated perspective into our revision of the standards for evidence. We have used the term “scaling up” to describe deliberate efforts to increase the impact of EBIs. These include efforts to broaden the populations (broadly defined to include whatever unit is targeted) to which the EBIs are delivered and the contexts in which they are implemented. As will become clear, we also added explicit standards to guide studies of the outcome of scale-up efforts. We recognize that while some preventive intervention research continues to be developed along a continuum, the progression along this continuum often involves numerous critical feedback loops to inform needed research at earlier phases of the research cycle. Often, effectiveness and scale-up research generate new questions that are later addressed in new efficacy or effectiveness studies.

We also recognize that much valuable prevention research does not follow the preventive intervention research cycle at all. For example, evaluations of the effects of interventions that are being widely implemented in a community but have not previously undergone randomized efficacy trials have great promise for identifying interventions with significant public health impact. In addition, important work conducted by economists, public policy analysts, and evaluation researchers often generates conclusive answers to questions about what works under what conditions, while focusing less on describing the intervention, testing the theoretical model underlying the intervention, or creating conditions conducive to subsequent scaling up. By organizing standards around the preventive intervention research cycle, our intent is not to question the value of efforts that are guided by these different frameworks. We expect, rather, that the standards articulated here may spur additional work to expand upon and complement these important works. For example, findings from evaluations of interventions that are delivered at scale may generate additional questions to be tested by new efficacy trials. Likewise, much can be learned about the factors influencing adoption, implementation, and sustainability of practices by conducting research on interventions that have not been developed according to the sequence implied in the preventive intervention research cycle.

Although we believe that continuing to recognize three stages of research is helpful, we recommend flexibility in following the sequence implied in the standards. Although the standards anticipate that certain questions will be answered at certain stages of the research cycle, a more flexible approach would allow researchers to take advantage of opportunities to answer important questions whenever they are available. For example, if the first trial of an intervention is a study of the intervention as it is practiced population-wide, later trials should seek to address questions that are more commonly raised in efficacy and effectiveness trials. The main focus should be on answering the questions implied for each stage in the research progression rather than rigidly adhering to the sequence at the expense of missed opportunities to contribute valued research.

The standards are loosely organized around the types of validity particularly critical for intervention research, as originally discussed in Cook and Campbell (1979) and elaborated on in Shadish et al. (2002):

- *Statistical conclusion validity* refers to the appropriate use of statistics to infer whether the intervention is related to the outcomes. Standards for statistical procedures to minimize threats to this type of validity are discussed primarily in the efficacy section.
- *Internal validity* pertains to inferences about whether the observed covariation between the intervention and the outcomes reflect a causal relationship. Standards for the design of the research and for minimizing threats to internal validity (such as differential attrition) are discussed primarily in the efficacy section.
- *Construct validity* pertains to inferences about higher order constructs that sampling particulars are thought to represent. Standards for describing the intervention and outcomes and for utilizing valid measures of them are discussed primarily in the efficacy section.
- *External validity* pertains to inferences about whether the observed cause-effect relationship holds over variation in persons, settings (including different places as well as different times), treatment variables, and measurement variables. Standards related to external validity are addressed primarily in the effectiveness and scale-up sections.

Consistent with the Flay et al. (2005) report, we provide sections on standards required for establishing that an intervention is efficacious, effective, and ready for scaling up. The standards are cumulative. That is, unless otherwise noted, all standards discussed for efficacy trials also pertain to trials in later stages of intervention development and testing. We also provide standards to guide decisions about the testing of outcomes of scale-up efforts. Although we retain unchanged standards, we discuss only modified and new standards, referring readers to the original Flay et al. (2005) work for a rationale for the unchanged standards. Standards that are taken with little or no modification from Flay et al. (2005) are indicated in bold in Tables 1, 2, and 3. Standards are considered to be modified if their placement in the efficacy, effectiveness, or broad dissemination section of the document has changed in this revision. In each section, we separate standards related to the intervention itself (e.g., description, theoretical basis, manuals, training, and technical assistance available) from the standards related to the conduct of studies of the intervention and standards related to reporting of research results. Tables 1, 2, and 3 summarize the new standards.

**Table 1:** SPR standards for efficacy

Number	Standards
1.	A statement of efficacy should be of the form that “Intervention X is efficacious for producing Y outcomes for Z population at time T in setting S.”
*Intervention description*	
**2.a.**	**The intervention must be described at a level that would allow others to implement/replicate it.**
2.b.	A clear theory of causal mechanisms should be stated.
2.c.	A clear statement of “for whom” and “under what conditions” the intervention is expected to be effective should be stated.
2.d.	The core components of the intervention and the theory relating these components to the outcomes must be identified and described.
2.e.	The anticipated timing of effects on theoretical mediators and ultimate outcomes must be described.
2.f.	It is necessary to characterize the research evidence supporting the potential that the intervention will affect outcomes that have practical significance in terms of public health impact.
*Measures and their properties*	
**3.a.**	**The statement of efficacy can only be about the outcomes that are measured and reported [Reporting Standard].**
3.b.	The quality and quantity of implementation must be measured and reported.
3.b.i	Precursors to actual implementation must be measured and reported.
3.b.ii	The integrity and level of implementation/delivery of the core components of the intervention must be measured and reported.
3.b.iii	The acceptance, compliance, adherence, and/or involvement of the target audience in the intervention activities must be measured and reported.
3.b.iv	Level of exposure should be measured, where appropriate, in both the treatment and control conditions.
*D*	*Document factors related to the quality and quantity of implementation.*
3.c.	Clear cost information must be reported [Reporting Standard].
*D*	*Report cost-effectiveness information.*
*D*	*Collect data on outcomes that have clear public health impact.*
***D***	***Measure potential side effects or iatrogenic effects.***
3.d.	There must be at least one long-term follow-up at an appropriate interval beyond the end of the intervention or, for ongoing interventions, beyond the implementation of the intervention.
**3.e.**	**Measures must be psychometrically sound.**
**3.e.i**	**Construct validity—Valid measures of the targeted behavior must be used, following standard definitions within the appropriate related literature.**
**3.e.ii**	**Reliability—Internal consistency (alpha), test–retest reliability, and/or reliability across raters must be reported.**
***D***	***Use of multiple measures and/or sources.***
**3.e.iii**	**Where “demand characteristics” are plausible, there must be at least one form of data (measure) that is collected by people different from the people who are applying or delivering the intervention. This is desirable even for standardized achievement tests.**
*Theory testing*	
4.	The causal theory of the intervention should be tested.
*Valid causal inference*	
**5.a.**	**The design must have at least one control condition that does not receive the tested intervention.**
**5.b.**	**Assignment to conditions needs to minimize bias in the estimate of the relative effects of the intervention and control condition, especially due to systematic selection, and allow for a legitimate statistical statement of confidence in the results.**
5.b.i	For generating statistically unbiased estimates of the effects of most kinds of preventive interventions, well-implemented random assignment is best.
5.b.ii	Publications should specify exactly how the randomization was done and provide evidence of group equivalence [Reporting Standard].
5.b.iii	Well-conducted regression discontinuity designs are second only to random assignment studies in their ability to generate unbiased causal estimates.
5.b.iv	For some kinds of large-scale interventions where randomization is not practical or possible, comparison time series designs can provide unbiased estimates of intervention effects.
5.b.v	Nonrandomized matched control designs rarely produce credible results. They should be used only under the certain conditions specified in text.
5.c.	The extent and patterns of missing data must be addressed and reported.
*Statistical analysis*	
**6.a.**	**Statistical analysis must be based on the design and should aim to produce a statistically unbiased estimate of the relative effects of the intervention and a legitimate statistical statement of confidence in the results.**
**6.b.**	**In testing main effects, the analysis must assess the treatment effect at the level at which randomization took place.**
**6.c.**	**In testing main effects, the analysis must include all cases assigned to treatment and control conditions.**
**6.d.**	**Pretest differences must be measured and statistically adjusted, if necessary.**
6.e.	When multiple outcomes are analyzed, the researcher must provide a clear rationale for the treatment of multiple outcomes.
*Efficacy claims*	
**7.a.**	**Results must be reported for every targeted outcome that has been measured in the efficacy study, regardless of whether they are positive, nonsignificant, or negative [Reporting Standard].**
**7.b.**	**Efficacy can be claimed only for constructs with a consistent pattern of nonchance findings in the desired direction.**
**7.c.**	**For an efficacy claim, there must be no serious negative (iatrogenic) effects on important outcomes.**
*Reporting*	
8.	Research reports should include the elements identified in the 2010 CONSORT guideline or a relevant extension of these guidelines.

*Note*: Desirable standards are shown in italics and denoted as “D” in the Number column. Bold indicates that the standard has been taken with little or no modification from Flay et al. (2005)

**Table 2:** SPR standards for effectiveness

Number	Standards
**1.**	**To claim effectiveness, studies must meet all of the conditions of efficacy trials plus the following standards.**
*Intervention description*	
**2.**	**Manuals and, as appropriate, training and technical support must be readily available**
*Generalizability*	
**3.a.**	**The degree to which findings are generalizable should be evaluated.**
**3.b.**	**The target population and setting as well as the method for sampling both populations and settings should be explained in order to make it as clear as possible how closely the sample represents the specified populations and settings that define the broad target for future dissemination [Reporting Standard].**
3.c.	The sample should contain a sufficient number of cases from each of the dimensions across which intervention effects are to be generalized to assess intervention effects in each subgroup.
*Population subgroups*	
4.	Statistical analysis of subgroup effects must be conducted for each important subgroup to which intervention effects are generalized.
*D*	*Statistical analyses testing group differences in the causal mechanisms should be provided if such differences have been proposed in the theory of the intervention.*
*Intervention tested*	
**5.a.**	**The intervention should be delivered under the same types of conditions as one would expect in the community institutions where such interventions are most likely to be situated during scale-up.**
5.b.	It is essential to compare the fidelity and quality of implementation/delivery of the core components of the intervention to that achieved in efficacy trials.
**5.c.**	**The recruitment, acceptance, compliance, adherence, and/or involvement of the target audience and subgroups of interest in the core intervention activities should be measured and reported.**
5.d.	Local adaptations to core components should be measured and reported.
5.e.	Factors related to the quality of implementation should be measured and reported.
5.f.	Convincing evidence that effects are not biased by investigator allegiance should be provided.
*D*	*In at least one effectiveness trial demonstrating desired outcomes, a researcher who is neither a current nor past member of the program developer’s team should conduct data collection and analysis.*
*Outcomes measured*	
**6.a.**	**The effects of an intervention must be practically important. Evaluation reports should report evidence of practical importance.**
6.b.	Cost-effectiveness information should be reported.
*D*	*Report cost-benefit information.*
*Effectiveness claims*	
7.	Effectiveness can be claimed only for intervention conditions, populations, times, settings, and outcome constructs for which the average effect across all effectiveness studies is positive and for which no reliable iatrogenic effect on an important outcome has been observed.
*Research to inform scale-up efforts*	
*D*	*Investigate the context, systems, and other factors that influence intervention adoption, quality implementation, and sustainability of the EBI.*

*Note*: Desirable standards are shown in italics and denoted as “D” in the Number column. Bold indicates that the standard has been taken with little or no modification from Flay et al. (2005)

**Table 3:** SPR standards for scaling up (broad dissemination)

Number	Standards
**1.**	**Only EBIs that have met all effectiveness criteria should be made available for scaling up.**
*Readiness for scaling up EBIs*	
*D*	*Prior to scaling up, it is desirable to assess readiness and to use the assessment in planning.*
*D*	*It is desirable for the scale-up effort to be implemented in the context of an organization development intervention to support the adoption, implementation, and sustained use of an EBI.*
**2.**	**Clear cost information and cost tracking and analysis tools that facilitate reasonably accurate cost projections and are practically feasible must be made available to potential implementers.**
*Materials*	
**3.**	**To be ready for scaling up, materials that specify the activities to be carried out and optimal methods of delivery must be available.**
*Training and technical assistance*	
**4.a.**	**To be ready for scaling up, training for implementing the core components of the intervention must be available.**
**4.b.**	**To be ready for scaling up, technical assistance must be available.**
*Fidelity assessment*	
**5.a.**	**Fidelity monitoring tools must be available to providers.**
5.b.	A system for documenting adaptations to core components should be in place prior to initiating the EBI. Adaptations should be addressed in ongoing technical assistance activities.
5.c.	A system to support regular monitoring and feedback using the available implementation monitoring tools should be in place.
*D*	*Normative data on desired levels of implementation keyed to the available implementation measures should be provided.*
*Improving the reach of the EBI*	
6.	A system should be in place to support planning and monitoring of client recruitment.
*Studying outcomes of scale-up efforts*	
7.	Scale-up efforts should be rigorously evaluated to ensure that at least the anticipated immediate effects are observed on outcomes of practical importance when the intervention is implemented on a population level.
*D*	*Before initiating rigorous scale-up research, it is desirable to conduct an evaluability assessment.*

*Note*: Desirable standards are shown in italics and denoted as “D” in the Number column. Bold indicates that the standard has been taken with little or no modification from Flay et al. (2005).

Standards of evidence are expected to change over time as methods develop and as Prevention Science and practice advance. For this reason, we also include standards that are desirable (labeled as such) though not essential given the current state of program development and evaluation. Just as we have upgraded many of the desirable standards of Flay et al. (2005) to required standards, we anticipate that many of our desirable standards may become required in the future as knowledge accumulates and methods advance.

## Standards for Efficacy

### Specificity of the Efficacy Statement [Reporting Standard]

1. *Standard: A statement of efficacy should be of the form that “Intervention X is efficacious for producing Y outcomes for Z population at time T in setting S.”*

Our first criterion pertains to the form of the efficacy statement. Because outcome research results are specific to the intervention actually tested, the samples (or populations), the point in time and settings from which they were drawn, and the outcomes measured, it is essential that conclusions from the research be clear regarding the intervention, population(s), time, and settings, and the outcomes for which efficacy is claimed. Subsequent studies should generalize beyond what is likely to be a fairly narrowly defined set of conditions in the efficacy trial.

### Intervention Description

- *Standard: The intervention must be described at a level that would allow others to implement/replicate it, including the content of the intervention, the characteristics and training of the providers, characteristics and methods for engagement of participants, and the organizational system that delivered the intervention.*
- *Standard: A clear theory of causal mechanisms (including identification of mediators as well as outcomes) should be stated.*
- *Standard: A clear statement of “for whom” and “under what conditions” the intervention is expected to be effective should be stated*.
- *Standard: The core components of the intervention (i.e., those hypothesized to be essential for achieving the advertised outcomes) and the theory relating these components to the outcomes must be identified and described.*

A clear and complete description of the intervention is necessary to guide practice, provide a basis for sound measurement of its implementation, and for replication. The standards for describing the intervention have been modified from Flay et al. (2005) to require that in addition to describing the intervention, an account of the theoretical mechanism through which the intervention is expected to influence the outcome is also provided. Chen (1990) and MacKinnon (2008) discuss the two components of this theoretical mechanism: The “action theory” corresponds to how the treatment will affect mediators, and the “conceptual theory” focuses on how the mediators are related to the outcome variables. To meet this standard, authors should provide an account of both the action and conceptual theories. Making these theories explicit should help the developer to identify the features of the intervention that are most central to the action theory. These features should be clearly identified as the “core” components of the intervention.^1^ These core components should be fully described.

The statement regarding the conditions under which the intervention is expected to be efficacious should clarify the populations, settings, times, and outcomes, or the “range of application” for the intervention. In so doing, the underlying assumptions about hypothesized similarities in the causal structure as well as anticipated limitations to application across populations, settings, times, and outcomes will be documented. This statement should also define the broad target for future dissemination if the intervention is demonstrated to be effective.

The level of detail included in these statements describing the intervention must be sufficient so that others would be able to replicate the intervention.

2.e. *Standard: The anticipated timing of effects on theoretical mediators and ultimate outcomes must be described.*

The intervention theory should also clarify when (relative to the intervention) the expected outcomes should be observed. The description of timing of outcomes should be based on an understanding of the developmental epidemiology of the targeted behavior. Is the intervention expected to influence the ultimate outcomes immediately (as, for example, adding lighting to a parking lot would be expected to influence theft from the lot), or is a lag anticipated (as, for example, encouraging attachment to school in elementary school children might be expected to reduce substance use during adolescence)?

2.f. *Standard: It is necessary to characterize the research evidence supporting the potential that the intervention will affect outcomes that have practical significance in terms of public health impact*.

Demonstrated public health impact is essential at a later stage of program development, but it should not be ignored at the efficacy stage. This standard requires that an argument be made connecting the observed outcomes to outcomes of practical significance. This connection can be accomplished by collecting and reporting data on such outcomes (e.g., number of subjects who stopped using tobacco as a result of the intervention; number of child abuse and neglect cases or crimes prevented, increases in number of high school graduates). If such outcomes are not available at the efficacy trial stage, a logical argument can be made to connect the available outcomes with outcomes of practical significance. For example, a study may collect data on known precursor of criminal activity such as low self-control or poor parental supervision. Making use of data collected by others that links these proximal outcomes to outcomes of practical importance, the researcher can characterize the potential of the intervention to produce practically meaningful outcomes.

### Measures and Their Properties

- *Standard: The statement of efficacy can only be about the outcomes (e.g., mediators as well as problem and well-being outcomes) that are measured and reported [Reporting Standard].*
- *Standard: The quality and quantity of implementation must be measured and reported.*
- *Standard: Precursors to actual implementation such as completion of training, practitioner-coach ratio, caseload, staff qualifications, and availability of necessary resources must be measured and reported.*
- *Standard: The integrity and level of implementation/delivery of the core components of the intervention must be measured and reported.*
- *Standard: The acceptance, compliance, adherence, and/or involvement of the target audience in the intervention activities must be measured and reported.*
- *Standard: Level of exposure should be measured, where appropriate, in both the treatment and control conditions.*

Implementation fidelity influences intervention outcomes (Durlak and Dupre 2008; Fixsen et al. 2005), and the quality of implementation of preventive interventions when delivered in “real-world” settings is often suboptimal (Gottfredson and Gottfredson 2002; Hallfors and Godette 2002; Ennett et al. 2003). Assessing implementation fidelity and quality is an important activity at all stages of development of an EBI (Allen et al. 2012), and tools to guide researchers in the reporting of implementation fidelity (e.g., Oxford Implementation Index, Montgomery et al. 2013a) are available. It is important to understand the extent to which the core components of the intervention can be varied and still achieve the desired effect and to document modifications that occur in the field. Although information on the quality and quantity of implementation will become more important in later stages of research, it is essential that such information be collected and reported in earlier trials that produce desired outcomes. This information will provide a benchmark against which implementation levels in later trials can be compared.

The level of implementation of the intervention is meaningful only in comparison to what is present in the comparison condition. Many interventions contain elements that are likely to be present in the comparison group as well as in the treatment group. For example, most drug treatment courts involve intensive probation, frequent judicial hearings, drug testing, and drug treatment. A lower dosage of these same components is likely to be part of usual service for a “treatment as usual” comparison group. Similarly, interventions that are related to the intervention of interest may be present in the control condition. It is important to document the differences between the services provided to the treatment and comparison groups while taking care not to allow the measurement itself to influence what is delivered in the control condition.

- *Desirable Standard: It is desirable to document factors related to the quality and quantity of implementation.*

It is desirable during the efficacy trial period to assess not only the quality and quantity of implementation (Efficacy *Standard 3.b.*), but also the factors that are likely to be related to variation in implementation. These factors include features of the intervention such as amount and type of training involved in implementing the intervention during the efficacy trial, the clarity of the intervention materials, the type of setting in which the intervention is tested, and external (social, economic, and political) forces in the larger community. Many efficacy trials are small in scope and would therefore not provide sufficient variability across different conditions of these factors to provide useful data without deliberate manipulation. Spoth et al. (2013) recommend embedding research on factors that are likely to be relevant in the dissemination stage (e.g., factors that might influence implementation quality when delivered in natural settings, factors that might influence communities’ decisions to select or adopt the intervention, etc.) into earlier stage research studies. Efficacy studies, for example, might randomly assign units to different levels of training and technical assistance, or to different levels of organization development assistance. Short of conducting this type of rigorous research on these factors, qualitative data on factors that are perceived to be related to implementation quality would provide a useful starting point for more thorough investigation into these factors at a later stage.

3.c. *Standard: Clear cost information must be reported [Reporting Standard].*

Flay et al. (2005, p. 167) included a standard stating that “clear cost information must be readily available” before an intervention is ready for scaling up. A discussion of the types of costs that should be included in the cost calculation was also provided. Glasgow and Steiner (2012) and Spoth et al. (2013) underscore the need for such information in community decisions to adopt and to sustain evidence-based practices later on. Prevention scientists can begin to pave the way for accurate cost tracking during the efficacy trial stage by documenting program costs.

Of course, assessing costs is not straightforward. There is currently no accepted standard to guide cost assessment, and considerable variability exists in what elements are included. Costs incurred during efficacy and effectiveness trials are likely to include significant costs related to conducting the research that are difficult to separate from the costs likely to be incurred by communities later adopting the intervention. Further, costs are likely to change over time as programs evolve. The Institute of Medicine recently held a workshop on standards for benefit-cost assessment of preventive interventions (http://www.iom.edu/Activities/Children/AnalysisofPreventiveInterventions/2013-NOV-18.aspx), and a recently formed SPR task force is studying this topic and will soon provide much needed guidance in this area. Prevention scientists should be guided by the forthcoming recommendations of these groups. In the meantime, investigators are encouraged to include in their cost reporting not only the cost of intervention materials and training, but also projected costs to the delivering organization, as discussed in Flay et al. (2005). These include:

- Nonresearch investments in delivery of staff training
- On-site time
- Facility, equipment, or resource rental and maintenance
- Reproduction of materials
- Value of volunteer labor and donated space and equipment
- Attendant delivery costs for consultants, clerical staff, and physical plants

Foster et al. (2007) provide a more detailed discussion of cost elements in prevention interventions and how they can be measured.

- *Desirable Standard: It is desirable to report cost-effectiveness information.*

Researchers do not usually estimate cost-effectiveness in efficacy trials. Even at the efficacy level, however, it is desirable to estimate cost-effectiveness (i.e., the cost of achieving the observed change in the outcome). This information will influence decisions to adopt the intervention at a later stage and so should be collected during earlier stages if possible.

- *Desirable Standard: It is desirable to collect data on outcomes that have clear public health impact.* 2 *Desirable Standard: It is desirable to measure potential side effects or iatrogenic effects.*

3.d. *Standard: There must be at least one long-term follow-up at an appropriate interval beyond the end of the intervention. For policy interventions whose influence is expected to continue for an indefinite period of time, evidence must be presented for a sustained effect of the policy for an appropriate interval after the policy was put in place.*

The positive effects of an intervention may diminish rapidly or slowly, or broaden and increase over time. Some interventions may demonstrate effects on problems that emerge later in development, such as substance use or abuse, sexual behavior, mental disorder, criminal behavior, or drunk driving (Griffin et al. 2004; Olds et al*.* 2004; Wolchik et al. 2002). Flay et al. (2005) recommended a follow-up interval of at least 6 months after the intervention but noted that the most appropriate interval may be different for different kinds of interventions. We believe that the 6-month time frame is a reasonable minimum time frame to demonstrate that effects observed at the end of the intervention do not dissipate immediately, but a more accurate picture of intervention effects requires that measurement time points coincide with the theory of timing of intervention effects specified in the intervention description (see above). This theory should be developed based on an understanding of the developmental epidemiology of the targeted behavior. For example, to demonstrate efficacy of a fifth grade intervention on outcomes that arise during adolescence, it is necessary to include measurement during adolescence rather than after 6 months. The causal theory linking the intervention to the ultimate outcomes (see Efficacy *Standard 2.e.*) should specify proximal outcomes and the expected timing of effects on them. The timing of measurement of both the proximal and ultimate outcomes should conform to this theory.

3.e. *Standard: Measures must be psychometrically sound*.

The measures used must either be of established quality, or the study must demonstrate their quality. Quality of measurement consists of construct validity and reliability.

3.e.i. *Standard: Construct validity—Valid measures of the targeted behavior must be used, following standard definitions within the appropriate related literature*.
3.e.ii. *Standard: Reliability—Internal consistency (alpha), test–retest reliability, and/or reliability across raters must be reported*.

- *Desirable Standard: It is desirable to use multiple measures and/or sources.*

3.e.iii. *Standard: Where “demand characteristics” are plausible, there must be at least one form of data (measure) that is collected by people different from the people who are applying or delivering the intervention. This is desirable even for standardized achievement tests.*

### Theory Testing

4. *Standard: The causal theory of the intervention should be tested.*

Although the primary emphasis in efficacy trials is on demonstrating that an intervention is efficacious for producing certain outcomes, understanding the causal mechanism that produces this effect will allow for greater generalization to the theory of the intervention rather than to the specific components of the intervention. For example, the knowledge that implementing a specific model of cooperative learning in a classroom increases achievement test scores is valuable. But knowledge that the mechanism through which this effect occurs is increased time on-task is even more valuable because it facilitates the development of additional interventions that can also target the same mediator. It is therefore important to measure the theoretical mediators that are targeted by the intervention.

As noted earlier (see Efficacy *Standard 2.d*.), the intervention theory involves both an “action theory” of how the treatment will affect mediators and a “conceptual theory” of how the mediators are related to the outcome variables. Both aspects of the intervention theory should be tested at the efficacy stage. Tests of the action theory probe the extent to which each core component influences the mediators it is hypothesized to move. The strongest of such tests would systematically “dismantle” the intervention into core components. That is, they would randomly assign subjects to conditions involving different core components and compare the effects of the different combinations on the hypothesized mediators. Of course, such designs are seldom feasible when the subjects are schools or communities. However, testing for intervention effects on the hypothesized mediators would provide a test of the action theory of the intervention as a whole.

Testing the conceptual theory involves analysis of mediating mechanisms. Despite recent advances in methods for testing mediational processes (e.g., MacKinnon 2008), these methods are not as well developed as are methods for testing casual effects of the intervention. Intervention theories often involve complex causal processes involving numerous mediators operating in a chain. Testing such complex causal chains in a rigorous fashion is not yet possible with existing tools (Imai et al. 2012). Even for simple theories involving only one mediator, it is not possible to test the theory underlying the intervention except in comparison with another theory. Available tools allow only for a rudimentary examination of the behavior of theorized mediating variables. Even so, such rudimentary tests can provide valuable information about which of the proposed mediators are both responsive to the intervention and correlated with the outcomes. Such information, although not constituting a strong test of the full intervention theory, at least provides information about which mechanisms are consistent with the stated theory. These tests should be conducted at the efficacy stage.

We caution that high-quality measurement of theoretical mechanisms will often be costly because it may require additional measurement waves between the intervention and the ultimate outcome as well as additional modes of measurement (e.g., observations). In some cases, the ultimate outcome may be decades in the future. Although measuring and testing causal mechanisms is critical to advancing science, in reality doing so may require trade-offs with the strength of the test of the effect of the intervention on the ultimate outcome. This trade-off creates tension that will have to be resolved over time as mediation analysis strategies improve and funding sources increase funding to allow for more rigorous testing of theoretical pathways through which interventions affect outcomes. In the meantime, this tension should be resolved in a way that preserves the integrity of the test of the intervention on the outcomes.

### Valid Causal Inference

- *Standard: The design must have at least one control condition that does not receive the tested intervention*.

The control condition can be no-treatment, attention-placebo, or wait-listed. Or, it can be some alternative intervention or treatment as usual (e.g., what the participants would have received had the new interventions not been introduced), in which case the research question would be, “Is the intervention better than a current one?”

5.b. *Standard: Assignment to conditions must minimize bias in the estimate of the relative effects of the intervention and control condition, especially due to systematic selection (e.g., self-selection or unexplained selection), and allow for a legitimate statistical statement of confidence in the results*.

Although there are many sources of bias in the estimation of causal effects, selection effects are the most serious and prevalent in prevention research. Researchers should assign units to conditions in such a way as to minimize these biases. Such assignment reduces the plausibility of alternative explanations for the causes of observed outcomes. This then increases the plausibility of causal inference about the intervention. The design and the assumptions embedded in the design must take into account exactly how people or groups were selected into intervention and control conditions and how influences on the treatment and control conditions other than the intervention might differ.

5.b.i. *Standard: For generating statistically unbiased estimates of the effects of most kinds of preventive interventions, well-implemented random assignment is best because it is most clearly warranted in statistical theory.*

Within the context of ethical research, it is necessary to use randomization whenever possible to ensure the strongest causal statements and produce the strongest possible benefits to society (Fisher et al. 2002). Many objections to randomization may be unfounded (Cook and Payne 2002). Randomization is possible in most contexts and situations. Gerber et al. (2013) provide numerous examples of RCTs conducted to test policies in diverse areas such as migration, education, health care, and disease prevention. The White House recently sponsored an event to encourage the use of RCTs to different policy options for social spending (http://www.whitehouse.gov/blog/2014/07/30/how-low-cost-randomized-controlled-trials-can-drive-effective-social-spending). In fact, the Cochrane registry (www.cochrane.org) contains over 700,000 entries on randomized trials. The level of randomization should be driven by the nature of the intervention and the research question. Randomization can be of individuals or of intact groups such as classrooms, schools, worksites, neighborhoods, or clinics (Boruch 2005; Gerber et al. 2013). Also, the timing of intervention can be randomly assigned to allow a short-term comparison of outcomes between the early and later intervention groups.

5.b.ii. *Standard: Reports should specify exactly how the randomization was done and provide evidence of group equivalence. It is not sufficient to simply state that participants/units were randomly assigned to conditions [Reporting Standard].*

Because correct randomization procedures are not always implemented or sometimes break down in practice, it is essential that the randomization process be described in sufficient detail so that readers can judge the likelihood that the initial randomization process was correct and has not broken down despite being initially implemented correctly. The description of the process should include details of exactly how cases were assigned to conditions and a discussion of the extent to which the assignment was well concealed, or could have been guessed at or tampered with. A “well-implemented” random assignment is one in which this possibility is judged to be small. Post-randomization checks on important outcomes measured prior to the intervention should be provided so that the pretreatment similarity of the experimental groups can be assessed.

Although random assignment is the strongest possible design for generating statistically unbiased estimates of intervention effects, and although perceived obstacles to random assignment are often not as difficult to overcome as initially anticipated, random assignment studies sometimes involve important trade-offs, especially in terms of generalization or statistical power. Researchers must sometimes rely upon fallback designs, hoping that the estimates of effects produced using these designs approach those that would be obtained through a random assignment study. There has been much debate about which designs should be considered as suitable alternatives when the trade-offs involved in random assignment are too costly. Fortunately, evidence from within-study comparisons of different alternatives versus random assignment have yielded invaluable information about which designs are likely to yield results most comparable to those obtained from random assignment studies. These within-study comparisons directly compare the effect size obtained from a well-implemented random assignment design with the effect size from a study that shares the same treatment group as the randomized study but has a nonrandomized comparison group instead of a randomly formed one. In these studies, the effects size obtained from the randomized arm of the study serves as a benchmark against which to compare the effect size obtained from the nonrandomized arm of the study.

Cook et al. (2008) summarize what has been learned from within-study comparisons and report results from 12 recent studies in an attempt to identify features of nonrandomized designs whose results match those from randomized designs most closely. This research identifies only two alternative designs, regression discontinuity designs and comparison time series designs, which reliably generate unbiased estimates of treatment effects. There are now a total of seven studies comparing regression discontinuity and experimental estimates at the regression discontinuity cutoff score and there are six comparing experimental and interrupted time series or comparison time series designs with a nontreatment comparison series. All point toward the causal viability of the quasi-experimental design in question. The third design considered in Cook et al. (2008) involves matched comparison group designs without a time series structure. In their paper, the results from these designs approach those of random assignment studies only under certain restrictive conditions—when the selection process happens to be completely known and measured well and when local intact comparison groups are chosen that heavily overlap with treatment groups on pretest measures of the outcome. Since then, somewhat conflicting claims have been made about the other quasi-experimental design features that promote causal estimates close to those of an experiment. The regression discontinuity, comparison time series, and matched group designs are described below, along with potential trade-offs involved with each. The trade-offs anticipated for randomized designs and the fallback design under consideration should be carefully weighed against each other when determining the strongest possible design for a given study.

Research on alternative quasi-experimental designs for evaluation studies is evolving quickly. The standards articulated here take advantage of the most rigorous research available to date, but we expect that as the field continues to evolve, additional alternative designs will be identified using the within-group comparisons strategy.

5.b.iii. *Standard: Well-conducted regression discontinuity designs are second only to random assignment studies in their ability to generate unbiased causal estimates.*

Regression discontinuity designs involve determining who receives an intervention based on a cutoff score on a preintervention measure. The cutoff score might be based on merit or need, or on some other consideration negotiated with the other research partners. For example, students with reading scores below the 25th percentile might be assigned to a tutoring intervention while the remaining students serve as a control, or communities whose per capita income level falls below a certain point might be targeted for certain services while those above the cut-point are not. The regression of the outcome on the assignment score is used to estimate intervention effects. Intervention effects are inferred by observing differences in the slopes and/or intercepts of the regression lines for the different groups. This design provides unbiased estimates of the treatment effects because, as in randomized studies, the selection model is completely known.

Cook et al. (2008) analyzed three within-study comparisons contrasting causal estimates from a randomized experiment with those from regression discontinuity studies. The regression discontinuity design studies produced comparable causal estimates to the randomized studies at points around the cutoff point. There are now four further studies in each of which the authors claim that the regression discontinuity and experimental results are similar at the cutoff. There is also one (Wing and Cook 2013) showing that when a pretest comparison function is added to the regular regression discontinuity (called a “comparison regression discontinuity function”), this mitigates the disadvantages of the regression discontinuity relative to the experiment. That is, regression discontinuity is more dependent on knowledge of functional forms, its statistical power is lower, and causal generalization is limited to the cutoff score (Shadish et al. 2002; Trochim 1984, 2000). Although Wing and Cook (2013) is the only relevant study with an experimental benchmark, its results indicate that a comparison regression discontinuity function can enhance statistical power almost to the level of the experiment, can help support conclusions about proper functional form that the nonparametric experiment does not need, and can attain causal conclusions away from the cutoff (and not just at it) that are similar to those of the experiment. So adding this particular comparison to the regression discontinuity function can significantly reduce the limitations of the regression discontinuity design relative to an experiment.

5.b.iv. *Standard: For some kinds of large-scale interventions (e.g., policy interventions, changes to public health law, whole-state interventions) where randomization is not practical or possible, comparison time series designs can provide unbiased estimates of intervention effects.*

Flay et al. (2005) included a standard recommending the use of interrupted time series designs for large-scale interventions where randomization was not feasible. The logic of this design is that the effect of an intervention can be judged by whether it affects the intercept or slope of an outcome that is repeatedly measured (Greene 1993; Nerlove and Diebold 1990; Shadish et al. 2002). For example, Wagenaar and Webster (1986) evaluated the effects of Michigan’s mandatory automobile safety seat law for children under 4 by comparing the rate of injuries to passengers 0–3 years old for the 4 years prior to enactment of the law and a year-and-three quarters after its enactment. Flay et al. (2005) pointed out that these designs could be strengthened by using comparison series in locations in which the intervention was not implemented, by using naturally occurring “reversals” of polices to test whether the outcome responds to both reversals and reinstatements of the policy, and by increasing the number of baseline time points.

Time series designs with only a single treatment group are rarely unambiguously interpretable because the effects of the intervention are often confounded with other events occurring at the same time.^3^ Often broad reforms are made in response to highly publicized, often emotionally laden, incidents. These incidents may result in numerous reforms that fall into place at roughly the same time, making it impossible to isolate the effects of only one of them using time series analysis. This is why all but one test of the similarity of experimental and ITS results deals with a comparison time series design rather than a single group interrupted time series design. Using the comparison time series design, the same outcome measures might be collected in a neighboring county or state or (in studies of school policy reform) in a grade level not affected by the reform. These designs, if well implemented, provide a means by which confounding effects due to co-occurring events can be ruled out. A nascent literature (reviewed in St. Clair et al. 2014) comparing the estimates from these comparison time series designs with those of randomized designs suggests that the comparison time series designs produce unbiased estimates of treatment effects (this assumes, of course, that there are few studies with conflicting results sitting in “file drawers”). Wagenaar and Komro (2013) encourage the use of these comparison time series designs for research evaluating public health laws and policies and discuss a number of design features (e.g., multiple comparison groups and multiple levels of nested comparisons, reversals, replications, examination of dose response) that can be used to further enhance these designs. These designs have broad utility for a wide variety of research needs including establishing theory-based functional forms of intervention effects over time (e.g., understanding the diffusion S-curves, tipping point transitions, and decay functions that often characterize effects when going to scale). We conclude that the comparison time series design, but not the single group interrupted time series design, can provide a useful alternative to randomized designs.

5.b.v. *Standard: Non-randomized matched control designs can rarely be relied on to produce credible results. They should be used only under the following conditions:(a) Initial group differences are minimized, especially by identifying intact comparison groups that are local to the treatment group and demonstrably heavily overlap with it on at least pretest measures of the outcome (Cook et al.* 2008 *); (b) the process by which treatment subjects select into the intervention group (or are selected into it) is fully known, well-measured, and adequately modeled (Diaz and Handa* 2006 *; Shadish et al.* 2008 *);or (c) the covariates used to model any group differences remaining after careful comparison group choice lead to no detectable pretest difference between the treatment and comparison groups in adequately powered tests. To this last end, it is desirable to explicate the selection process and use it to choose covariates or, where this is not possible, to include as many covariates as possible that tie into multiple domains.*

Early reviews of within-study comparisons (Glazerman et al. 2003; Bloom et al. 2005) concluded that estimates of effects from studies using common strategies for equating groups (e.g., matching, analysis of covariance, propensity scoring, selection modeling) are often wrong. A well-known example is the research on hormone replacement therapy for women where prior nonrandom trials suggested positive effects but a large randomized trial found harmful effects (Shumaker et al. 2003). A more recent summary of within-study comparisons comes to a slightly more optimistic conclusion about the value of nonrandomized matched comparison group designs. Cook et al. (2008) summarized results from nine within-study comparisons of random assignment versus matched comparison groups. Some but not all of these matched comparison estimates were similar to the estimates obtained in the randomized arm of the study. Cook et al. (2008) described the very specific conditions under which nonrandomized matched designs produce similar results to randomized designs.

First, studies in which researchers designed the study beforehand to identify an intact comparison group that was “likely to overlap with the treatment group on pretest means and even slopes” (Cook et al. 2008, p. 736) resulted in comparable experimental and nonexperimental study effect size estimates. For example, Bloom et al. (2005) evaluated the National Evaluation of Welfare-to-Work Strategies. One part of this evaluation reported on five sites in which a comparison group from a randomized trial conducted in a different job training center was used as a matched comparison group, but the comparison training centers were located in the same state (or the same city in four of the five sites), and the measurement was taken at the same time as the measures for the subjects in the job training sites that were the focus of the evaluation. No matching of individual cases was conducted, but the careful selection of intact groups from similar locations and times resulted in pretest means and slopes that did not differ between the treatment and comparison groups. Conversely, when intervention and comparison sites were from different states or even different cities within a state, the groups were not at all equivalent and differences could not be adjusted away. Cook et al. (2008) concluded that the use of intact group matching, especially using geographic proximity as a matching variable, is a useful strategy for reducing initial selection bias.

The second condition under which effects sizes from nonrandomized matched comparison group designs matched those from randomized studies involved treatment and nonrandomized comparison groups that differed at pretest but where the selection process into treatment was known and modeled (Diaz and Handa 2006). An example of this type of study comes from an evaluation of PROGRESA in Mexico. In this study, eligible villages were randomly assigned to receive the intervention or not, and eligible families in treatment villages were compared with eligible families in control villages on outcomes. Eligibility for the intervention was based on scores on a measure of material welfare, both at the village level and at the individual family level within village. The design identified villages that were too affluent to be eligible for PROGRESA. These villages were clearly different than the villages that participated in PROGRESA, but the selection mechanism that resulted in some villages and families being selected into the study and others not was completely known and measured. Once the same measure of material welfare that had determined eligibility for PROGRESA was statistically controlled, selection bias was reduced to essentially zero.

These conditions—intact group matching and complete knowledge of the selection process—are rare. It is not yet clear when nonrandomized matched comparison group designs that do not meet these conditions will yield valid results, regardless of the technique used for statistical adjustment. However, the likelihood of bias is reduced when initial group equivalence is inferred from adequately powered no-difference results on multiple, heterogeneous baseline measures that include at least one wave of pretest measures of the main study outcome. This is the criterion currently advocated by the What Works Clearinghouse of the Institute for Educational Sciences (http://ies.ed.gov/ncee/wwc/).

In nonexperimental studies, the choice of data analysis technique is not very important for reducing selection bias. Direct comparisons of ordinary least squares and propensity score matching methods have not shown much of a difference to date (Glazerman et al. 2003; Bloom et al. 2005; Shadish et al. 2008; Cook et al. 2009), though the latter is theoretically preferable because it is nonparametric and requires demonstrated overlap between the treatment and comparison groups on observed variables. More leverage for reducing selection bias comes from (a) preintervention theoretical analysis of the selection process into treatment—or even direct observation of this process—so as to know which covariates to choose, (b) selecting local comparison groups that maximize group overlap before any covariate choice, and (c) using a heterogeneous and extensive collection of covariates that, at a minimum, includes one or more pretest waves of the main study outcome (Cook et al. 2009).

The evidence to date suggests that randomized studies are less vulnerable to bias than nonrandomized studies, but that regression discontinuity and comparison time series designs may be suitable alternatives to randomized studies. However, it bears repeating that a poorly implemented randomized design is as likely to yield biased results as a nonrandomized study. Randomization can be subject to tampering. But even when executed faithfully, randomized trials often suffer from differential attrition across study groups, which often reduces group equivalence and renders the study results ambiguous. Therefore, we caution against any process for identifying efficacious interventions that identifies effective interventions based on the initial study design without carefully considering the quality of implementation of the design. We also provide the following standard to guide reporting of randomization procedures (above) and analysis and reporting of study attrition:

5.c. *Standard: The extent and patterns of missing data must be addressed and reported.*

Analyses to minimize the possibility that observed effects are significantly biased by differential patterns of missing data are essential. Sources of missing data include attrition from the study, from particular waves of data collection, and failure to complete particular items or individual measures. Missing data is particularly troubling when the extent and pattern of missing data differs across experimental conditions. Differences across conditions in the nature and magnitude of attrition or other missingness can bias estimates of intervention effects if they are not taken into account. Note that differential measurement attrition can occur even when the rates of attrition are comparable across groups.

Schafer and Graham (2002) discuss methods of analyzing data in the face of various kinds of missingness. One common strategy is to impute missing data based on the data that are available. Appropriate application of these imputation methods requires assumptions about the pattern of missingness, however, and these assumptions are often not justified in practice. The required assumption is that missing data are “missing at random,” which means that there is no discernible pattern to the missingness once measured variables are controlled. If this assumption cannot be met (as is often the case), sensitivity tests should be conducted to probe the likely impact that missing data might have on the estimates of the intervention effect (Enders 2011; Imai 2009; Muthen et al. 2011).

### Statistical Analysis

- *Standard: Statistical analysis must be based on the design and should aim to produce a statistically unbiased estimate of the relative effects of the intervention and a legitimate statistical statement of confidence in the results*.
- *Standard: In testing main effects, the analysis must assess the treatment effect at the level at which randomization took place.*

In many contexts in which prevention researchers carry out their work, the participants belong to naturally occurring groups that often must be taken into account when conducting statistical tests. For example, if a researcher is testing a drug prevention curriculum in third grade classrooms, the fact that the students belong to the classrooms means those student responses may not be independent of other students in the same classroom, and this has an important impact on the validity of the statistical tests. Often, researchers will randomize at a higher level (e.g., the school) but analyze the data at a lower level (e.g., individuals). Doing so almost always results in a violation of the assumption of the statistical independence of observations. Even small violations of this assumption can have very large impacts on the standard error of the effect size estimate (Kenny and Judd 1986; Murray 1998), which in turn can greatly inflate the type I error rate (e.g., Scariano and Davenport 1987). In these situations, analysts must conduct analyses at the level of randomization and must correctly model the clustering of cases within larger units (Brown 1993; Bryk and Raudenbush 1992; Hedeker et al. 1994; Zeger et al. 1988). For example, if an intervention is delivered at the clinic level (e.g., some clinics deliver a new intervention, others do not), then clinics should be randomly assigned to conditions, and the statistical analyses must take into account that patients are nested within clinics.

6.c. *Standard: In testing main effects, the analysis must include all cases assigned to treatment and control conditions (except for attrition—see above).*
6.d. *Standard: Pretest differences must be measured and statistically adjusted, if necessary.*
  That is, when differences between groups on pretest measures of outcomes or covariates related to outcomes are observed, models testing intervention effects should incorporate these pretest values in a manner that adjusts for the preexisting differences*.*
6.e. *Standard: When multiple outcomes are analyzed, the researcher must provide a clear rationale for the treatment of multiple outcomes, paying close attention to the possibility that conclusions may reflect chance findings.*

There is no consensus on the best way to handle this issue in prevention research. However, an expert panel recently convened by U.S. Department of Education Institute of Educational Sciences explored ways of appropriately handling multiple comparisons (Schochet 2007). This panel recommended that outcomes be prioritized to reflect the design of the intervention and that confirmatory analyses be conducted to test global hypotheses within the main domains identified as central to the study’s hypotheses. For example, a program might include a tutoring component aimed at improving academic performance and a social skills curriculum aimed at improving social competency skills. Schochet (2007) recommends that multiple measures of academic performance (e.g., teacher reports of academic competence, grade point average, standardized reading, and math scores) be combined into one scale to test the hypothesis that the program influences academic performance and that multiple measures of social competency (e.g., goal setting, decision-making, and impulsive control) be combined into a second scale to test the hypothesis that it influences social competency skills. The report recommends against testing each of the multiple measures as a separate outcome.

Our standard does not require that researchers follow this advice, but rather that they attend carefully to potential misinterpretations due to the analysis of multiple correlated outcomes and provide a clear rationale for the treatment of multiple outcomes.

### Efficacy Claims—Which Outcomes?

- *Standard: Results must be reported for every targeted outcome that has been measured in the efficacy study, regardless of whether they are positive, nonsignificant, or negative [Reporting Standard].*
- *Standard: Efficacy can be claimed only for constructs with a consistent pattern of nonchance findings in the desired direction. When efficacy claims are based on findings from more than one study, efficacy can be claimed only for constructs for which the average effect across studies is positive.*

Note first that this standard pertains to *constructs* rather than to measures of constructs. For studies reporting findings for multiple measures of the same construct, an omnibus test that corrects for alpha inflation must confirm a nonchance effect in the desired direction (see *Standard 6.d.*).

This standard can be met either within one study or through replication. Replication has two main purposes in Prevention Science: To rule out chance findings and to demonstrate that results obtained in one study are robust to variations in time, place, and certain implementation factors. The latter are generalizability issues that are most appropriately addressed in effectiveness trials. Before an intervention can be judged to be a suitable candidate for effectiveness trials, though, the possibility that positive results were due to chance must be minimized.

Flay et al. (2005) called for at least two different studies of an intervention, each meeting all of the other efficacy standards, before an intervention could be labeled as “efficacious.” This standard is consistent with recent calls in Psychology for more direct replication studies to rule out chance findings. Pashler and Harris (2012) note that replication studies that test the same experimental procedure are extremely rare in psychological research, but they are essential to the conduct of science. They calculate that more than a third of published positive results are likely to be erroneous, even when researchers set low alpha levels (e.g., .05).^4^ Further, “conceptual” replications, in which researchers vary aspects of the intervention or the research operations, do not help to rule out chance findings because failures to replicate in such studies are too easily attributed to the variations tested rather than to the possibility that the earlier results were due to chance.

Flay et al. (2005, p. 162) recognized that exact replication in which the same intervention is tested on “a new sample from the same population, delivered in the same way to the same kinds of people, with the same training, as in the original study” is rare, and suggested that “flexibility may be required in the application of this standard …until enough time passes to allow the prevention research enterprise to meet this high standard.”

Time has passed. The prevention research enterprise appears no closer to reaching this high standard for replication to rule out chance findings, and funding agencies are no more likely today to fund replications simply to verify the results of an earlier study than they were 10 years ago. When replication studies are conducted, they are much more likely to be for the purpose of testing variations in the intervention or of generalizing results to different settings or populations than for ruling out chance findings. Although the accumulation of positive results from this type of replication study does eventually rule out chance findings, we regard these studies as generalizability studies most appropriate for interventions that have met all of the efficacy standards.

How should chance be ruled out at the efficacy stage? Chance can be ruled out in a single study if the magnitude of the intervention effect observed in a well-designed and well-conducted trial is so large that it is extremely unlikely to have arisen by chance given a true null hypothesis. That is, highly improbable significance levels lend confidence to the conclusion that the results are unlikely to be due to chance. For example, using Pashler and Harris’ (2012) reasoning, significant findings at the .005 level would translate into an actual error rate of approximately 5 %. Differences of this magnitude from a single trial should suffice to rule out chance.

When intervention effects from a single efficacy trial are not large enough to confidently rule out chance, one or more additional trials are needed. Data from these additional trials, when combined together with the first trial, must achieve a sample size large enough to test whether findings for the combined dataset exceed chance levels. Also, in order to rule out chance at the efficacy level, it is important that all experimental units be exposed to the same intervention rather than to different variants of the intervention, as is often the case in subsequent trials of an intervention. As noted by Flay et al. (2005), efficacy trial replications should be “exact” replications (Hunter 2001) in which the same intervention is tested on a new sample of the same population, delivered in the same way by the same kinds of people, with the same training, as in the original study, or “scientific” replications (Hunter 2001) in which all aspects are exactly replicated except that the study samples comes from similar populations rather than the exact same population (such as is likely in a multisite evaluation of an intervention). Judgments about the similarity of the population should be made on the basis of the program developer’s statement of the range of application of the intervention (see *Standard 2.c.*).

7.c. *Standard: For an efficacy claim, there must be no serious negative (iatrogenic) effects on important outcomes.*

### Reporting

8. *Standard: Research reports should include the elements identified in the 2010 CONSORT guideline or a relevant extension of these guidelines.*

Several of the standards articulated above are standards for reporting about prevention research. For example, Standard 2.a. requires that the intervention “be described at a level that would allow others to implement/replicate it,” and Efficacy Standard 7.a. states that “results must be reported for every targeted outcome that has been measured in the efficacy study.” Most of the standards pertain to research methods that should be fully described in reports of the research. Unfortunately, reporting of interventions tested and the methods used to evaluate them is often suboptimal, even in our best journals (Grant et al. 2013), and this often makes it difficult to judge the quality of the evidence from potentially important prevention trials. Research reports are often brief, omitting or inadequately reporting important information. Incomplete and inaccurate reporting results in underuse of the research.

Incomplete reporting is a problem in other disciplines as well. This has led to the development of numerous guidelines for reporting of research across different fields, the most well known of which is the Consolidated Standards of Reporting Trials (CONSORT) guideline, which has been recently updated (Schulz et al. 2010). CONSORT is intended to facilitate the writing of transparent reports by authors and appraisal of reports by research consumers. It consists of a checklist of 25 items related to the reporting of methods, including the design, who the participants were, how the sample was identified, baseline characteristics of the participants on key variables, how the sample size was determined, statistical methods used, participant flow through the study (including attrition analysis), the delivery, uptake, and context of interventions, as well as subsequent results.

The CONSORT guideline was developed by biomedical researchers to guide reporting of health-related clinical trials. It is therefore not broad enough to cover all reporting issues relevant for reporting of Prevention Science research. An extension of the CONSORT guideline has been proposed to guide transparent reporting of implementation, including how intervention implementation is adapted in the trial (Glasgow and Steiner 2012). Other extensions of the CONSORT guideline have been tailored to certain types of research common in Prevention Science (e.g., cluster randomized trials, Campbell et al. 2012). Nevertheless, the available guidelines are insufficient to cover many types of research in Prevention Science.

A new CONSORT extension for randomized controlled trials in social and psychological research is under development (Gardner et al. 2013) and is likely to address many of the special reporting issues in Prevention Science research (Mayo-Wilson et al. 2013. Indeed, this effort is addressing several aspects of intervention research discussed in the earlier SPR standards of evidence (Flay et al. 2005), such as active ingredients or mechanisms of interventions, interventions that operate and outcomes that are analyzed at multiple levels (e.g., individual, family, school, community), intervention implementation, the role of context (e.g., effectiveness versus efficacy; site differences in a multisite randomized trials), subgroup analysis, and intervention adaptation. This effort is well underway, with systematic reviews of guidelines and reporting practices (Grant et al. 2013), a modified Delphi process, and formal expert consensus meeting completed (Montgomery et al. 2013a; see project website: www.tinyurl.com/consort-study), and is likely to produce highly relevant reporting guidelines for Prevention Science. CONSORT guidelines cover only randomized trials. For nonrandomized designs, an appropriate reporting guideline should be used, such as the TREND statement (Des Jarlais et al. 2004) for behavioral and public health interventions. We note that this guideline would benefit from updating to reflect advances in causal designs described in Efficacy Standard 5.b.

We encourage SPR to collaborate in the development of standards for reporting of randomized controlled trials in social and psychological research and to create a task force, or join in with other groups, to work on refining these standards to make them broadly applicable to a wider range of research designs. As these more specific guidelines become available, the standard should be changed to reflect their availability.

## Standards for Effectiveness

The scope of Prevention Science has expanded in the past 10 years away from simply demonstrating efficaciousness and toward translation of efficacious practices into wider use in the population (Spoth et al. 2013). While it remains important to develop and test new prevention approaches, the field must simultaneously demonstrate that observed outcomes from efficacious interventions generalize to a wider range of populations, settings, and times and that these effective interventions can be translated into regular practice in communities (Glasgow and Steiner 2012).

Effectiveness trials seek to increase the generalizability of findings from efficacy studies. As noted earlier, study results can be generalized only to the intervention actually tested, the process through which it was implemented, the samples (or populations), the time and settings from which they were drawn, and the outcomes measured. Most efficacy studies are limited in terms of generalizability because the intervention tested is one in which the developer has exerted substantial control, the population is often carefully selected to be amenable to the intervention, and the setting is likely to be one that is conveniently accessed by the researcher. As such, efficacy trials tell us little about the effect of the intervention under “real-world” conditions or in “natural” settings (Flay 1986). Efficacy trials also often have small sample sizes and are thus incapable of demonstrating effectiveness across different population subgroups.

Increasing generalizability of intervention effects is therefore a primary goal of effectiveness trials. This goal is most often met through one or more replication studies. Effectiveness trials can also set the stage for later scale-up efforts by exploring factors that are likely to influence later intervention adoption, quality implementation, and sustainability. As recommended by Spoth et al. (2013), we include desirable standards related to preparing for scale-up efforts.

1. *Standard: To claim effectiveness, studies must meet all of the conditions of efficacy trials plus the following standards.*

### Intervention Description

2. *Standard: Manuals and, as appropriate, training and technical support must be readily available.*

### Generalizability

- *Standard: The degree to which findings are generalizable should be evaluated*.
- *Standard: The target population and setting as well as the method for sampling both populations and settings should be explained in order to make it as clear as possible how closely the sample represents the specified populations and settings that define the broad target for future dissemination (see Efficacy Standard 2.c.). Settings must be community institutions that are the target for future dissemination [Reporting Standard].*
- *Standard*: *The sample should contain a sufficient number of cases from each of the dimensions across which intervention effects are to be generalized to assess intervention effects in each subgroup.*

Effectiveness seeks to answer the question: Are positive effects observed in efficacy trials robust to the variations in the intervention, populations, time, settings, and outcomes measured that are likely to be encountered when the intervention is implemented more broadly than it was in the efficacy trial? The first step in answering this question is to specify the most important dimensions across which the intervention effects should be generalized. This determination should be made during the intervention development stage. A clear statement of “for whom” and “under what conditions” the intervention is expected to be effective should guide decisions about the most important population groups, settings, outcomes, and variations in intervention to be tested (see Efficacy *Standard 2.c.*).

In order to test robustness across different dimensions, it is important to collect data that include a sufficient number of cases from each important dimension. This allows for sufficient statistical power to test for differences across these dimensions. Ideally, the sample would be a random sample from the population to which the intervention effects are to be generalized, stratified by the important dimensions identified for generalizability testing. However, probability samples (at least of individuals) are rarely used in Prevention Science because tests of interventions generally require that the subjects be located in a limited geographical area. Purposive samples including sufficient variability on the important dimensions are more feasible, but such samples must be carefully described so that their characteristics can be compared with those of the intended population. As was recommended in describing the sample and sampling methods for efficacy trials, we recommend that the CONSORT guidelines be used for reporting these aspects of the study.

Below we provide standards for testing intervention robustness across specific dimensions that are most likely to be important for generalizability: population subgroups, variations in intervention, and measured outcomes.^5^

### Population Subgroups

Population subgroups are often defined by membership in a category defined by gender, race or ethnicity, social class, or risk level. For large-scale interventions targeting entire populations, important subgroups may include types of communities (e.g., urban, suburban, rural) or types of institutions (e.g., alternative schools, regular schools).

4. *Standard: Statistical analysis of subgroup effects must be conducted for each important subgroup to which intervention effects are generalized.*

Demonstration of robust effects across important population subgroups can be accomplished by testing for group differences within a single study that has sufficient power to support such tests, or by testing for significant intervention effects in separate studies containing samples that are homogeneous with respect to the dimension. In the former, statistical tests should demonstrate that group differences in the intervention effect do not exceed chance levels. If they do exceed chance levels, subgroup analyses should be conducted to identify the source and nature of the differences. Additional guidance on subgroup analysis in Prevention Science is provided in Supplee et al. (2013).

- *Desirable standard: Statistical analyses testing group differences in the causal mechanisms should be provided if such differences have been proposed in the theory of the intervention (see Efficacy Standard 2.b.).*

Some intervention theories predict that the intervention will operate differently for different subgroups, or that the intervention must be varied in order to achieve similar effects across different population subgroups. Planned variations in the intervention to enhance effectiveness within a specific population are best treated as new interventions and tested using the standards for efficacy described earlier. Tests of differing causal mechanisms can be conducted as part of effectiveness trials if they are specified in advance and if the relevant mediating variables have been measured.

The same limitations discussed in the efficacy section for testing causal mechanism (see *Standard 4*) apply here. These tests are not likely to yield definitive results but can at least provide information about group differences in proposed mediators that are both responsive to the intervention and correlated with the outcomes. Such information can be useful in guiding efforts to refine the intervention to improve effectiveness across groups.

### Intervention Tested

- *Standard: The intervention should be delivered under the same types of conditions as one would expect in the community institutions where such interventions are most likely to be situated during scale-up (e.g., by teachers rather than research staff).*
- *Standard: It is essential to compare the fidelity and quality of implementation/delivery of the core components of the intervention to that achieved in efficacy trials.*

Examination of robustness to variation in implementation quality is important in effectiveness trials because they are expected to have greater variation than in efficacy trials. It is important to generate data from these trials about the extent to which weaker implementation quality diminishes effects and to understand the extent to which modifications applied in the field influence outcomes. This information will be critical as the intervention is scaled up (Glasgow and Steiner 2012). The level of fidelity and quality achieved in efficacy trials is a useful benchmark against which to compare such data from effectiveness trials.

5.c. *Standard: The recruitment, acceptance, compliance, adherence, and/or involvement of the target audience and subgroups of interest in the core intervention activities should be measured and reported.*

Consistent with the goal of effectiveness trials to probe generalizability across population subgroups, an emphasis on subgroup analysis has been added to this standard. Data on subgroup differences in the take-up of an intervention will help to explain any observed differences in the effectiveness of the intervention for these groups.

5.d. *Standard: Local adaptations to core components should be measured and reported.*

Local adaptations are modifications made by implementing organizations and communities that are not part of the intervention as described by the developer. Treatment of such adaptations is discussed at greater length in the section on scale-up efforts.

5.e. *Standard: Factors related to the fidelity and quality of implementation should be measured and reported.*

This standard, identified as desirable during the efficacy trial period, is essential at the effectiveness trial stage. To repeat, factors related to the quality of implementation include features of the intervention such as amount and type of training and technical assistance available and clarity of the intervention materials, characteristics of the organization adopting the intervention, the type of setting in which the intervention is tested, and external (social, economic, and political) forces in the larger community. Local adaptations are often made to interventions at the effectiveness stage in an attempt to increase adoption or implementation. Such adaptations should be documented (see above) and the effects of the adaptations on implementation fidelity should be noted (Allen et al. 2012). Effectiveness trials are likely to include meaningful variation on at least some of these potentially important factors. Interpretations of correlational analyses relating these factors to levels of implementation fidelity and quality are likely to be ambiguous because factors related to the quality of implementation are likely to be related to other important predictors of the outcomes, and these differences often remain uncontrolled in analysis. Such analyses can nevertheless provide information about factors related to variability in implementation fidelity and quality that will be valuable during scale-up efforts. Similar analyses of the association between these factors and study outcomes may also be helpful, despite inherent ambiguities in interpretation.

5.f. *Standard: Convincing evidence that effects are not biased by investigator allegiance should be provided.*

- *Desirable Standard: In at least one effectiveness trial demonstrating desired outcomes, a researcher who is neither a current nor past member of the program developer’s team should conduct data collection and analysis.*

One of the most important features of the intervention trial that must be tested for bias and generalizability is the involvement of the developer of the intervention. There is a continuum of developer control over intervention implementation and research. In early stages of testing, the developer’s team generally controls intervention materials, training, staff selection and supervision, feedback on implementation, data collection, and data analysis. During effectiveness trials, developer control over staff selection and supervision and (sometimes) training and quality control is loosened. Many prevention scientists believe that before an intervention is judged to be ready for scaling up, control over data collection and analysis should also be passed to an independent investigator.

Flay et al. (2005) stated that “it is desirable eventually to have some effectiveness trials that do not involve the developer—to establish whether interventions are sustained and still effective when the developer is not involved” (p. 162). This standard is seldom met in Prevention Science, even though such studies are clearly feasible. Several independent evaluations have yielded results similar to those reported by the developer (see, for example, Gardner et al. (2006) and Hutchings et al. (2007) as well as the Menting et al. (2013) systematic review of Incredible Years trials that reported no effect of developer involvement on the effect size). On the other hand, the past decade has also witnessed a disturbingly high rate of failures to replicate when independent evaluation teams conduct studies of prevention interventions and practices that had met all criteria for effectiveness.^6^

This challenge has been recognized more broadly in the social sciences. Pashler and Wagenmakers (2012)) reported on the status of replication in psychological science. A review of psychological research published in psychology journals with the highest 5-year impact factors showed that the percentage of findings that are replicated varied remarkably depending upon whether the replication research team was the original research team or not. Nearly all findings (92 %) from the original research team replicated, compared with 65 % from an independent team (Makel et al. 2012). Similarly, Petrosino and Soydan (2005) reported on results from a review of randomized field trials in criminology and criminal justice. They reported that effect sizes from trials conducted by program developers/creators were more than twice the size of effect sizes from trials conducted by others.

The reason for this pattern of findings is not clear. Nosek et al. (2012) summarized research that documented a number of common practices^7^ researchers engage in that, although sometimes justifiable, are known to increase the proportion of false results. These practices may be more common among original research team members and those with high allegiance to this team, for whom the desire to replicate is likely to be strongest (Ioannidis 2012). Of course, the observed pattern of more positive findings from research conducted by original research teams is equally consistent with a fidelity explanation: Program developers are likely to understand their interventions better and be more attuned to variations in implementation quality that might diminish intervention effectiveness. They are likely to attend more and demand higher adherence to intervention fidelity standards.

Unless they can rule out investigator allegiance as a factor contributing to higher effect sizes, designer-controlled programs of research will be weakened with respect to the effect sizes that might be expected in real-world applications when the developer is absent. We have therefore added a standard that requires that evidence be presented before scaling up that effects are not biased by investigator allegiance.

The most straightforward ways to rule out designer effects are through replication of positive outcomes by an independent research team or, ideally, through evidence from meta-analysis that developer involvement is not related to effect size across multiple studies. However, requiring independent replication prior to scale-up at present would severely limit the number of interventions available for scaling up because so few have met this high bar. This requirement would add additional time to the already lengthy time needed to for establishing the efficacy and subsequent effectiveness of evidence-based prevention interventions, which can involve a decade or more of development and evaluation. The challenge is one of weighing the realities of what is needed to test a preventive intervention with the need to move effective practices into routine practice. The goal should be to maximize scientific rigor in establishing their efficacy and effectiveness while also reducing the time needed to establish their readiness for dissemination and implementation.

The goal of making independent evaluations of preventive interventions routine is arguably too distant at this time to justify establishing a requirement that these evaluations be completed prior to scaling up. Instead, we hope to elevate awareness of the issue of investigator allegiance and to suggest that addressing this issue will increase the scientific integrity and credibility of our field. Our standard instead requires attention to the issue of investigator allegiance and a discussion of the steps that have been taken to minimize it, adjust for it, or bracket its potential effects. We also include independent replication as a desirable standard in the near term while the field builds consensus about this issue as well as the capacity to more routinely carry out these evaluations. We hope to encourage developers to move toward partnering with different research teams to conduct independent evaluations of their programs and to attune funding agencies to the importance of these endeavors. We anticipate that this desirable standard can become a required standard in the future.

### Outcomes Measured

- *Standard: To be considered effective, the effects of an intervention must be practically important. Evaluation reports should report evidence of practical importance*.

The standards for efficacy require only that a plausible argument be presented for why the intervention has the potential to affect outcomes that have practical significance in terms of public health impact. Such information is critical at the effectiveness trial stage because it will influence decisions to adopt the intervention later. Assessing multiple outcomes that are likely to tap into the priorities of different audiences makes research more likely to be translated into practice (Glasgow and Steiner 2012). Therefore, effectiveness trials must collect data on and report results for outcomes that would be considered important to the broader community that would be served by such interventions (e.g., crimes committed, high school graduation and employment rates, reports of child maltreatment, health outcomes). Note that these outcomes may include intermediate outcomes (e.g., hypertension, obesity, physical inactivity, diabetes as risk factors for cardiovascular disease) when such intermediate outcomes are widely accepted, based on empirical evidence that they are major risk factors for the subsequent outcome. Such empirically supported intermediate outcomes are commonly accepted as meaningful in public health research and can be applied productively to assess the effectiveness of interventions for the prevention of behavioral health outcomes.

As a general rule, the practical significance of the outcome can be expressed in terms of the combination of strength of effect and size of population affected. However, metrics of practical significance are likely to differ depending on the nature of the EBI. For EBIs targeting individuals or relatively small aggregates such as families, it is customary to use effect size calculations. EBIs that target entire populations may produce outcomes of practical significance even if the magnitude of the effect on individuals is small. These complexities need to be considered when describing the practical significance of the outcome.

6.b. *Standard: Cost-effectiveness information should be reported.*

This standard, identified as desirable in the efficacy stage, is elevated to a standard at the effectiveness stage because this information will be necessary before scale-up efforts are undertaken (Glasgow and Steiner 2012)*.* See earlier discussion regarding cost elements (Efficacy *Standard 3.c.*).

- *Desirable Standard: It is desirable to report cost-benefit information.*

Cost-benefit analysis assigns a monetary value to the measure of effectiveness and compares the cost of the intervention to the savings achieved through its implementation. For example, a preschool program may produce large benefits to society through later crime reduction. Although this information on longer term benefits of prevention interventions is seldom available, such information would likely to be a powerful tool at the adoption and sustaining stages of scale-up.

### Effectiveness Claims

7. *Standard: Effectiveness can be claimed only for intervention conditions, populations, times, settings, and outcome constructs for which the average effect across all effectiveness studies is positive and for which no reliable iatrogenic effect on an important outcome has been observed.*

This standard requires summarizing outcomes across available effectiveness studies. The earlier SPR standards required that effectiveness could be claimed only for outcomes for which there are “similar effect sizes in the preponderance of evidence from effectiveness trials” (Flay et al. 2005, p. 166). We believe that most policymakers care more that the intervention will produce a positive effect on an outcome of practical importance than they do about the exact magnitude of the effect. It is also less likely that different trials will produce similar effect sizes than that they will replicate a positive effect on an outcome.

Consistent with the recommendations of a recent SPR task force on replication in Prevention Science (Valentine et al. 2011), we recommend that data synthesis techniques, including meta-analysis or analysis of combined individual-level datasets, be used to characterize the average effect across effectiveness studies, and that effectiveness be claimed only for those outcome constructs for which the average effect size across all tests of that construct be in the positive direction, and that any outcome construct in the undesired direction can be attributed to chance. Only studies that have been carried out in “real-world” conditions (e.g., conditions that match the intended target for future dissemination—see Efficacy *Standard 2.c.*) should be included in this average. Such a pattern of findings supports a claim of effectiveness.

It is also important to characterize the extent to which the effectiveness claim is robust to the variations in intervention conditions, population characteristics, settings, and times that have been tested. An effectiveness claim for a subgroup, setting, or intervention condition can be supported by either showing that the overall positive outcome does not differ significantly across these conditions (e.g., by reporting a nonsignificant homogeneity statistic in a meta-analysis), or by showing that the average effect size for each condition is positive with no reliable iatrogenic effects on important outcomes.

As Prevention Science advances and more studies of specific EBIs become available, the importance of data synthesis across studies will become more critical in establishing effectiveness. Although models are available (Perrino et al. 2013), agreed-upon structures and mechanisms to facilitate the sharing of individual-level datasets for combination and data synthesis do not currently exist. We encourage SPR to create a task force to work on developing standards to guide the sharing of individual-level datasets for combination and data synthesis.

### Research to Inform Scale-up Efforts

Relatively little rigorous research has been conducted related to the processes and systems through which EBIs are adopted, implemented, and sustained on a large scale. Spoth et al. (2013) lay out an ambitious agenda for research to inform scale-up efforts. Scholars of dissemination and implementation research recommend that activities be undertaken throughout the planning, development, and evaluation of an intervention to increase its dissemination and implementation potential (Rabin and Brownson 2012). Spoth et al. (2013) also argue that it is important to embed research on these important questions into effectiveness trials of the EBIs so that essential information is available to guide later scale-up efforts. Below we list several research questions, mostly identified by Spoth et al. (2013), that we believe can be most feasibly studied in ongoing effectiveness trials of a particular EBI.

Research on factors influencing adoption decisions:

- What are the channels of information through which stakeholders learn about the EBI?
- What are the key market, organizational, and other factors influencing adoption decisions?
- What are the incentives/disincentives for EBI adoption by various stakeholders?
- What decision-making tools do stakeholders use in selecting an EBI?
- How are cost and other economic data used in the decision to adopt an EBI?

Research on implementation fidelity and quality:

- What are the characteristics of providers who are most likely to implement the EBI?
- What aspects of implementation fidelity and quality are related to outcomes?
- What are the most effective delivery systems for the EBI in different settings?
- What are the effects of different training and technical assistance methods on implementation fidelity and quality?
- How do amount, type, and mode of delivery of training and technical assistance affect implementation quality?
- What are the key factors that influence participation in EBIs? And what are the best strategies for enhancing participation?
- What are the relative contributions of EBI core components and how do specific adaptations affect outcomes?
- What is the cost of implementing the intervention?

Research on sustainability:

- What management, motivation, organization, training, and technical assistance factors for organizations and communities lead to greater sustainability?
- What funding models and financing strategies are most conducive to sustainability?
- What policies are most conducive to stable funding streams?

Research on these features might involve descriptive research on consumer preferences carried out during the site recruitment phase or during implementation. It might also involve examining how natural variation in characteristics of interventions or communities varies with intervention implementation quality. For example, a study might examine differences in implementation quality by potentially important intervention features (e.g., delivery agents, training quantity) or by measures of community capacity (although interpretation of findings would have to recognize that such associations are likely to be confounded with other important predictors of implementation quality). Stronger tests of moderating effects of intervention features could be obtained by embedding a study to test the effect of deliberate manipulation of the intervention feature on measures of implementation quality or sustainability. For example, effectiveness trials might randomly assign units to different levels of training and technical assistance, to different levels of organization development assistance, to different intervention delivery agents (e.g., nurses versus nurse practitioners; peers versus teachers), or different modes of delivery (e.g., online training or in-person training).

Clearly, much research is needed to support the translation of EBIs into broader use. We agree that effectiveness research represents an important opportunity to address these questions. In addition to the required standard for reporting on factors related to implementation quality in effectiveness trials (Effectiveness *Standard 5.e.*), we offer the following desirable standard promoting research on factors related to adoption, implementation, and sustainability of EBIs:

- *Desirable Standard: It is desirable in effectiveness trials to investigate the context, systems, and other factors that influence intervention adoption, quality implementation, and sustainability of the EBI.*

## Standards for Scaling Up of Evidence-Based Interventions

Much attention has been directed in the past decade toward challenges confronted in the scale-up of prevention interventions. This scale-up process entails translation of interventions that have been demonstrated to be effective when tested on a limited scale into standard practice on a broad (population-level) scale. The goal of such scale-up efforts is to achieve population impacts on important outcomes through sustained, high-quality implementation of EBIs. Reaching this goal requires awareness of the multiple factors that influence intervention implementation in natural contexts. Successful implementation requires careful attention to a wide range of factors that can influence the quality and sustainability of the implementation. It depends upon the confluence of features of the intervention, characteristics of the organization adopting the intervention, and external (social, economic, and political) forces in the larger community (Fixsen et al. 2005).

A recent report from an SPR task force on type 2 translational research (Spoth et al. 2013), while recognizing that this new “translation science” is still in its infancy, offered a framework for thinking about the phases of translation from research to practice, summarized research related to these different phases, and set ambitious agendas for both practice (e.g., building community infrastructures to support scale-up of evidence-based practices) and research to advance the science of translation.

Spoth et al. (2013) described activities, strategies, and processes concerning four phases of translational functions:

- Preadoption phase: addressing intervention, consumer, provider, and organizational characteristics with scale-up feasibility assessments or dissemination/marketing plans
- Adoption phase: attending to adoption decision-making factors and processes
- Implementation phase: integrating quality implementation procedures and processes into service systems or settings
- Sustainability phase: institutionalizing or maintaining over the long term and expanding reach

They summarized research on the features of both the intervention and the adopting organizations and communities that appear related to successful outcomes at each phase. Noting the paucity of research on most of these features, they identified research questions that still need to be answered in order to advance a science of translation, and they discussed a variety of research designs and methods that might be applied to answer these questions. They also noted that little rigorous research has been conducted to assess the outcomes of scale-up efforts.

The work of this SPR task force on type 2 translational research provides a useful framework for refining the SPR standards of evidence related to the scaling up of EBIs. The earlier standards (Flay et al. 2005) recognized that successful dissemination was a function of a complex interplay between the intervention developer and the adopting community, and identified key features of an intervention that needed to be in place in order to increase the likelihood that typical service providers or others could implement it effectively. Below we elaborate on these features, incorporating findings from recent research on features of interventions that are related to successful adoption, implementation, and sustained implementation of EBIs.

In addition to attending to these features of interventions, it is necessary to create a strong organizational and community infrastructure for translational success. An intervention may be ready to be scaled up in terms of the research supporting its effectiveness and (if appropriate) the materials, training, and technical assistance available to communities, but it will not be adopted, implemented, or sustained in the absence of local capacity to adopt, implement, and sustain its use. The scientific research base for understanding the most important features of organizations and communities that predict their success in adopting, implementing, and sustaining evidence-based practices is not as well developed as is the research on features of interventions that promote these same outcomes, but enough consensus exists to provide a basis for establishing preliminary standards for practice related to community and organizational infrastructure (Aarons et al. 2012; Spoth et al. 2013). We anticipate that the work of the SPR task force on type 2 translational research will encourage new research to further clarify important organizational factors that can later be incorporated into a refined set of standards to guide organizations and communities in developing capacity to adopt, implement, and sustain effective interventions.

The literature on characteristics of adopting organizations and communities is far reaching. Some of this work has implications for actions at the federal and state levels to improve community capacity for adopting, implementing, and sustaining EBIs. We stopped short of incorporating standards related to efforts to improve capacity in general (such as improving dissemination of knowledge about EBIs or creating funding structures) because the scope of our work is more narrowly defined. Our objective is to update the earlier effort to “determine the most appropriate criteria *for prevention programs and policies* to be judged efficacious, effective, or ready for dissemination” (Flay et al. 2005, p. 152). We defined efforts to assess organizational or community readiness for adoption of a particular EBI, to encourage high-quality implementation, and to prepare organizations or communities for sustained high-quality delivery of particular EBIs as within our scope, while more general activities that are not directly connected to particular EBIs were defined as outside of our scope.

### Readiness for Scaling Up EBIs

The standards regarding readiness for scaling up are not standards of “evidence” in the sense used elsewhere in this document. The standards provided to guide efficacy and effectiveness trials pertained to the features of the research evidence that are required in order to label an intervention as efficacious or effective. The standards described below are features of the *intervention* or of the *organization or community adopting the intervention* rather than of the evidence about the consequences of implementing the intervention. These features of interventions or environments have been shown to be related to adoption, implementation, or sustainability of the intervention. These standards are intended to provide practical guidance to scale-up efforts.

Four recent reviews summarize much of the relevant research on the characteristics of interventions and environments that are related to high-quality delivery of EBIs. Durlak and DuPre (2008) summarized findings from 81 studies of prevention and health promotion for children and adolescents that contained data on factors affecting the implementation process. Fixsen et al. (2005) reviewed studies concerning implementation from a much broader set of domains (including agriculture, business, child welfare, engineering, health, juvenile justice, manufacturing, medicine, mental health, nursing, and social services). They located 377 “significant implementation” articles, 22 of which reported the results of experimental analyses or meta-analyses of implementation variables. A recent volume on dissemination and implementation research in health (Brownson et al. 2012 and especially a chapter in that edited volume on the role of organizations in that process (Aarons et al. 2012) and the Spoth et al. (2013) review described earlier provide comprehensive reviews of the most recent research on factors related to adoption, implementation quality, and sustainability of EBIs. Note that the existing literature on characteristics related to high-quality delivery of EBIs focuses mainly on smaller scale interventions that usually involve education or provision of other services. The standards reflecting this evidence base therefore include a disproportionate focus on characteristics of service providers, materials, training, and technical assistance. The characteristics related to high-quality implementation of larger scale EBIs such as laws or broad policies are not as well documented but are likely to involve some of these same activities.

1. *Standard: Only EBIs that have met all effectiveness criteria should be made available for scaling up.*

#### Preadoption and Adoption Phases

Several of the activities recommended by Spoth et al. (2013) aimed at encouraging adoption of EBIs are community-level supports and practices that occur well-before a particular EBI has been identified. Decisions about how best to make information about EBIs available to a community and the establishment of epidemiological data sources to guide community decision-making about which EBIs would suit the needs of the community are outside of the scope of our work (see above). Here we provide guidance on three activities that can be undertaken with respect to a particular EBI: assessing community readiness for the EBI, embedding the EBI in an appropriate organization development intervention to strengthen community capacity, and providing clear information about the costs of the EBI.

- *Desirable Standard: Prior to scaling up, it is desirable to conduct an assessment of factors that are likely to impede or facilitate adoption and successful implementation of the EBI and the capacity of the community or organization to implement the EBI in a high-quality fashion. This assessment can use an available psychometrically sound community assessment tool, or it can take the form of original research to describe local conditions that are likely to influence adoption, implementation, and sustainability. The results of the assessment should be utilized in planning efforts during the adoption phase.*

There is general consensus that an assessment of the organization or community’s capacity to implement EBIs in a high-quality fashion, conducted prior to the decision to adopt, would be helpful for uncovering likely obstacles to implementation that could be addressed prior to adoption or for identifying strengths that could improve implementation (Spoth et al. 2013). Aarons et al. (2012) summarize research on characteristics of organizations that increase their likelihood of adopting and implementing EBIs. Among the most important characteristics that encourage a climate supportive of implementing EBIs is the presence of a strong leader who communicates a vision that includes the use of evidence to guide practice and encourages adherence to that vision. Such leadership encourages positive attitudes toward adopting EBIs (Aarons et al. 2012). Prior to scaling up an intervention, an assessment of factors such as the leadership potential in the community should be undertaken.

Fixsen et al. (2005) reviewed several available scales designed to measure different aspects of community readiness to implement EBIs, including attitudes about EBIs, motivational readiness, community resources, staff attributes, organizational climate, and stages of community readiness. Although good psychometric properties have been reported for these scales, we know of no rigorous research that has tested the validity of the scales for predicting improved implementation quality or sustainability of an intervention.

Related to community readiness assessment is the recommendation that “practice-oriented research” can help to enhance community conditions conducive to adoption, implementation, and sustained use of EBIs (Spoth et al. 2013). This type of research is highly collaborative and focused on answering practical questions. Such research, conducted early in a scale-up effort, could provide invaluable information about the local context, culture, decision-making processes, and history that could guide local adaptations of noncore aspects of the intervention and help with planning to remove likely barriers to recruitment, acceptance, participation, and high-quality implementation. Such research, tailored to the specific community, could serve the same purpose as a more formal community readiness assessment.

We suggest that the use of some form of community readiness assessment is desirable, assuming predictive validity can be established. At present, very little research is available to guide the use of data from such assessments in practice. That is, although interventions are currently being developed and tested to encourage readiness factors such as strong leaders (Aarons et al. 2012), much research is needed in this area. We anticipate that future research will provide clearer evidence of the value of such assessments.

- *Desirable Standard: It is desirable for the scale-up effort to be implemented in the context of an organization development intervention to support the adoption, implementation, and sustained use of an EBI.*

This standard recognizes that characteristics of the community organization(s) adopting the EBI are likely to be at least as important as characteristics of the intervention in determining success. Often, preliminary efforts are required to shore up community infrastructure to increase the likelihood that the intervention will be implemented in a high-quality fashion and sustained over time. Emerging research suggests that EBIs of the future should include an organization development component to increase its adoption, implementation, and sustainability. For example, Glisson et al. (2010) demonstrated in a randomized trial that multisystemic therapy (MST) is more effective when implemented in the context of an organization development intervention that focuses on improving leadership and community agency culture and climate than when MST is implemented alone.

Community partnership models are another form of organizational development intervention that have been shown to enhance selection, adoption, and sustained use of EBIs (Fagan et al. 2009; Spoth et al. 2011; Spoth and Greenberg 2011). This research has demonstrated the success of two different models of community partnerships/coalitions, both of which draw upon existing community resources and involve a high degree of community input in decision-making about the selection and implementation of EBIs. Because the community factors influencing sustainability of EBIs are likely to be dynamic, the model used to develop community infrastructure needs to incorporate continuous assessment of the local context and ongoing decision-making to maintain the match between the EBI and the community (Chambers et al. 2013).

2. *Standard: Clear cost information and cost tracking and analysis tools that facilitate reasonably accurate cost projections and are practically feasible must be made available to potential implementers.*

Providing clear cost information was identified as a standard beginning at the efficacy stage. Such information should be refined throughout the testing of the EBI so that it provides an accurate estimate of the cost of the intervention as delivered at scale, under natural conditions of service implementation by community settings. Spoth et al. (2013) suggest that cost tracking and analysis tools that facilitate reasonably accurate cost projections and are practically feasible be made available to communities. As noted earlier, standards do not currently exist to guide decisions about which cost elements should be included in cost accounting. Recommendations from an Institute of Medicine workshop and an SPR task force should be available soon. In the meantime, we recommend that the cost elements discussed under Efficacy *Standard 3.c.* be included.

#### Implementation Phase

Flay et al. (2005) discussed standards related to the materials, training, and technical support that must be in place before an intervention is ready to be taken to scale. Since these standards were articulated, additional research has clarified which aspects of these materials, training, and technical assistance appear most critical to ensuring adoption, high-quality implementation, and sustainability. We therefore offer refinements to these initial standards to better align them with current research findings.

### Materials

3. *Standard: To be ready for scaling up, materials that specify the activities to be carried out and optimal methods of delivery must be available. Materials should include a clear statement of the conditions necessary to implement the intervention, including characteristics of the setting, and qualifications of intervention providers. If appropriate, standardized audiovisual aids, reproducible materials, and lists of materials needed should be included.*

Some interventions do not involve materials. For interventions that do, research recommends the use of manuals that include reproducible materials, provide audiovisual aids, provide lists of materials to be used, and specify activities to be carried out because such materials are related to higher scores on measures of implementation quality than interventions lacking such structured materials (Gottfredson and Gottfredson 2002). Interventions that are attractively packaged and easy to use and that utilize teaching methods familiar to the provider are also more likely to be adopted and implemented (Rohrbach et al. 2006). Intervention materials that include a clear statement of the conditions necessary to implement the intervention, including characteristics of the setting, qualifications of intervention providers, content, and methods are also more likely than less explicit materials to be implemented with higher quality (Fixsen et al. 2005). These findings suggest that standardization and structure are important features of intervention materials.

The findings summarized in Durlak and Dupre (2008), however, suggest that adaptability and flexibility are key features of the intervention related to implementation quality. An adaptable intervention is one that can be modified to fit the needs of the organization and community potentially implementing the intervention. Rabin and Brownson (2012) identify reinvention or adaptation as essential to the success of dissemination and implementation efforts and suggest that such adaptation is likely to lead to “at least equal intervention effects as shown in the original efficacy or effectiveness trial” (p. 35).

Durlak and Dupre (2008) note the apparent contradiction between the importance of fidelity and adaptability in the research on implementation quantity and suggest that the apparent contradiction is resolved by recognizing that both qualities can coexist in an intervention. The challenge is to find the right mix of standardization and adaptability that maintains the integrity of the intervention while allowing for flexibility at the local level concerning adaptability to conditions. How to do this is a challenge that is currently the subject of debate and research in the prevention field (see, for example, the ongoing study by the CDC Foundation to learn how community-based organizations adapt EBIs and whether or not those adaptations make the interventions more or less effective; CDC Foundation 2013). Until more definitive information is available to guide this process, we propose (consistent with Rabin and Brownson 2012) that intervention developers provide a clear statement about what aspects of the intervention are considered core components that cannot be altered, and which are more flexible. Standards related to the description of the intervention have been revised to require identification of core components (Efficacy *Standard 2.d.*), and a new standard has been added to encourage testing of core intervention components (Efficacy *Standard 4*).

### Training and Technical Assistance

- *Standard: To be ready for scaling up, training for implementing the core components of the intervention must be available. This training must include demonstration of the new practices, ample opportunity for practicing the new skills, and feedback on performance of the new skills. A clear statement concerning which aspects of the intervention can be locally adapted should be included in the training. The training should be consistent with the level of training that was found to produce high-quality implementation in effectiveness trials* (see Effectiveness *Standard 5.e.*)*.*
- *Standard: To be ready for scaling up, technical assistance must be available for the EBI. This technical assistance must be proactive and must provide ongoing support for additional teaching while the practitioner is engaged in practice activities, direct assessment and feedback on performance, and emotional support.*

While much of the research on the provision of technical assistance concerns interventions involving direct services, it is likely that high-quality implementation of larger scale interventions such as passage of new laws or changes to broad policies often requires some type of technical assistance to those charged with implementing the changes. The nature of the technical assistance is likely to differ depending on the type of intervention. Research on training and technical assistance suggests that although initial training is usually required to provide knowledge about the intervention, training by itself seldom improves the quality of implementation (Fixsen et al. 2005). Training that provides information only and “one-shot” training with no follow-up are especially unlikely to produce high-quality implementation. However, training that includes demonstration of the new practices, ample opportunity for practicing the new skills, and feedback on performance of the new skills is related to implementation quality (Fixsen et al. 2005). Durlak and DuPre (2008, p. 338) add that training “should not only help providers develop mastery in specific intervention skills, but also attend to their expectations, motivation, and sense of self-efficacy, because the latter can affect their future performance in and support of the new innovation.” In addition, training should include a clear statement of which components are considered “core” components and should not be altered, and which aspects of the intervention can be adapted to achieve a better fit with local conditions.

Experimental research has shown that the addition of high-quality technical assistance to training produces higher quality implementation than does training alone. Such technical assistance maintains providers’ motivation and commitment, improves their skill levels where needed, and supports local problem-solving efforts (Durlak and DuPre 2008). In particular, technical assistance that incorporates on-the-job coaching has been repeatedly shown to be related to the quality of performance (Fixsen et al. 2005). Such coaching provides additional teaching while the practitioner is engaged in practice activities, direct assessment and feedback on performance, and emotional support. Also, as many providers do not self-identify the need for technical assistance, the provision of technical assistance should be proactive rather than on demand (Spoth et al. 2013).

### Fidelity Assessment

- *Standard: Fidelity monitoring tools must be available to providers. These tools should include measures of precursors to actual implementation such as completion of training, acceptable practitioner-coach ratio, acceptable caseload, availability of colleagues with special skills, and availability of necessary resource; integrity and level of implementation/delivery of the core components; and acceptance, compliance, adherence, and/or involvement of the target audience in the intervention activities.*
- *Standard: A system for documenting adaptations to core components should be in place prior to initiating the EBI. Adaptations should be addressed in ongoing technical assistance activities.*
- *Standard: A system to support regular monitoring and feedback using the available implementation monitoring tools should be in place.*

- *Desirable Standard: Normative data on desired levels of implementation keyed to the available implementation measures should be provided.*

Fidelity to the core components of the intervention is essential. When implementation quality is monitored, implementation quality improves. Durlak and DuPre (2008) summarized results of meta-analyses showing that interventions that monitored implementation obtained effect sizes two to three times larger than interventions that reported no monitoring.

Monitoring of implementation is important at each stage of research. Standards guiding the development and use of fidelity measures and the reporting of fidelity information were presented in the efficacy and effectiveness sections. During scale-up efforts, the same implementation quality and fidelity measures should be utilized, and a system to support regular monitoring and feedback using these tools should be developed.

While simply measuring implementation and providing feedback to implementers will improve fidelity and quality, fidelity assessments are likely to be most helpful if the level of implementation observed during the scale-up attempt is compared with a benchmark level that has been shown in prior research to yield desired effects. Prior effectiveness trials will have generated data necessary to provide this critical feedback.

### Improving Reach of the EBI

6. *Standard: A system should be in place to support planning and monitoring of client recruitment. Planning should include a careful assessment of local barriers to participation and identification of strategies to overcome these barriers. Recruitment efforts should be monitored on an ongoing basis and planning should be renewed as often as necessary to ensure high participation rates.*

Scale-up efforts aim to achieve population impacts on important outcomes through sustained, high-quality implementation of EBIs. Failure to engage a large enough fraction of the target population (e.g., persons, organizations, institutions intended to participate) is a major impediment to the scaling up of EBIs (Spoth et al. 2013). Failure to reach those segments of population most at risk for experiencing problems (e.g., inner city neighborhoods, schools with weak leadership) and traditionally underserved communities (e.g., rural communities) is especially problematic for prevention efforts. The most effective methods for achieving high levels of participation of the populations most likely to benefit from the intervention are likely to differ across type of interventions and communities, but careful attention to recruitment can improve the reach of the intervention.

#### Sustainability Phase

Many of the same factors that influence adoption decisions and implementation quality also influence sustainability of interventions (Spoth et al. 2013). In general, the challenge in achieving sustained high-quality implementation of EBIs involves integrating into usual practice in the community or organization the training, technical assistance, implementation monitoring, and feedback for the new practices for which standards have already been discussed. As most of these topics have already been incorporated into standards in the previous sections, no additional standards have been identified at this time for the sustainability phase.

However, we note that sustained use of EBIs can be facilitated by a community partnership or coalition, as discussed above, that continually reviews and addresses problems as they arise. The two community partnership/coalition models discussed previously (PROSPER and Communities that Care) have both been demonstrated in rigorous research to sustain implementation of EBIs. Spoth et al. (2013) discuss alternative models that, for example, establish state-level technical assistance systems, data systems that provide regular feedback on implementation quality, organization development activities, and structures for addressing leadership turnover. Lastly, they discuss the importance of establishing policies and mechanisms to sustain funding for EBIs.

### Standards for Studying Outcomes of Scale-up Efforts

7. *Standard: Scale-up efforts should be rigorously evaluated to ensure that at least the anticipated immediate effects are observed on outcomes of practical importance when the intervention is implemented on a population level. Consistent with the standards for efficacy and effectiveness trials, the research design for these studies must be “the strongest possible given the nature of the intervention, research question, and institutional framework within which the intervention/research occurs. The design must also be well executed, and any remaining threats to causal inference, or alternative explanations for observed effects, should be addressed” (Flay et al.* 2005 *, p. 157). Randomized controlled trials, comparison time series designs, or regression discontinuity designs are preferred, although creative adaptations to these designs are likely to be required.*

Flay et al. (2005) included standards for providing evaluation tools to practitioners so they can evaluate the effects of their efforts, but stopped short of providing guidance for more rigorous evaluation of scale-up efforts. The assumption was that once an intervention cleared all hurdles related to demonstrating efficacy and effectiveness, it would be likely to produce similar effects upon scale-up if implemented with fidelity. Recent failures to replicate initial strong findings in the prevention field (referenced earlier) raise doubts about this assumption. A similar phenomenon has been observed in biomedical research: Ioannidis (2005) compared effect sizes from subsequent high-quality studies of highly cited clinical research studies published in three major general clinical journals. He found that the initially reported effects from a third of these influential studies were subsequently either contradicted or found to be substantially smaller than initially reported. This research suggests that even effects that are regarded as firmly established are often called into question in subsequent studies.

Many possible explanations of this “shrinking effects” phenomenon exist, including initial publication and time-lag bias favoring the more rapid and prominent publication of “positive” findings, as suggested by Ioannidis (2005). Evolving treatment and counterfactual conditions might also explain the phenomenon. Interventions often change over time, presumably to meet evolving client needs. Similarly, the services to which clients would be exposed in the absence of an EBI change over time. Services with overlapping goals are frequently developed and implemented. This process creates an ever-changing “treatment as usual” condition against which the intervention of interest should be compared. This point was recently made in response to evidence from a national evaluation of Head Start, a major early childhood program, indicating that effects of the program were small and faded out more rapidly than anticipated (Puma et al. 2012). Ludwig and Phillips (2008) commented that alternative early childhood programs have been developed and evaluated since the initiation of Head Start that are far more cost effective than Head Start. Perhaps the availability of more effective alternatives renders the program of interest less desirable to implement on a large scale. Ludwig and Phillips (2008) also pointed out that the evidence supporting Head Start’s long-term effects is from studies of children who participated in Head Start in 1980 or earlier. While the quality of the Head Start program has improved since that time, so has the quality of the environments to which youth would be exposed in the absence of the program. “Which environment is improving faster in this horse race is unclear” (Ludwig and Phillips 2008, p. 261).

It is premature to draw conclusions about which mechanisms underlie the phenomenon of changing effect sizes over time, and they are likely to vary by the intervention in question as well. But it seems clear that we cannot necessarily expect effect sizes to stay constant over time and that continued assessment of outcomes is needed after scale-up in order to monitor changes in effect sizes over time and reevaluate prevention options. How should these scale-up efforts be studied?

Studies of scale-up efforts often focus on implementation quality rather than outcomes (Chamberlain et al. 2008; Forgatch and DeGarmo 2011). When they do measure outcomes, the research designs often do not meet high standards of scientific rigor (Bloomquist et al. 2013; Schroeder et al. 2011; Winokur Early et al. 2012). Some dissemination attempts have been studied using randomized trials, however (Rohrbach et al. 2010). Spoth et al. (2013), in the context of a discussion of appropriate research designs for answering questions about factors related to intervention adoption, implementation, and sustainability, discussed challenges to using traditional research designs appropriate for efficacy and effectiveness trials to study scale-up efforts. In particular, they suggested that it might be problematic to withhold EBIs from a control group in a large-scale effort whose aim is to provide effective programming to an entire population. They discussed alternatives to the traditional randomized controlled trial design, including “roll out” designs in which organizations are randomly assigned to an implementation start-up time rather than to a permanent treatment or control condition. While such designs are not useful for documenting sustained effects of interventions (because control cases eventually receive the treatment), they are an excellent choice for demonstrating that at least the initial outcomes of an intervention are consistent with those found in earlier studies. State-of-the-art methods have also been offered for analyzing data from randomized controlled trials to maximize what is learned from scale-up efforts. For example, intent-to-treat analyses often neglect important variation in the effect of an intervention across individual characteristics, context, and time. Examining such variation may be particularly important in scale-up efforts that reach a broader swath of the population. Brown et al. (2008) provide guidance in how data generated from multilevel randomized designs can be analyzed to estimate heterogeneity of effects. Curran et al. (2013) also discuss models for blending design components of effectiveness and implementation research that may be particularly useful in the studying of scale-up efforts.

Other rigorous designs such as comparison time series and regression discontinuity may also be appropriate designs for many scale-up efforts. If an intervention is initiated all at once for an entire population, if archival measures of relevant outcomes (e.g., arrest rates, child maltreatment cases, substance abuse-related emergency room visits) are available for several time points before and after the start-date, and if a second population can be identified for which the same data points are available but the intervention is not planned, comparison time series is ideal. If a new service is to be provided on a limited basis to individuals or families meeting certain criteria, if a scale can be developed to assess these criteria, and if the implementing organization agrees to use a cut-point on the scale to assign subjects to receive services or not, a regression discontinuity design may be appropriate. In short, rigorous research designs that effectively rule out alternative explanations for observed findings is as critical in evaluations of scale-up efforts as it is in efficacy and effectiveness trials. Rigorous research designs can be employed creatively to study these efforts.

- *Desirable Standard: Before initiating rigorous scale-up research, it is desirable to conduct an evaluability assessment to evaluate the likelihood that the evaluation of the intervention in scale-up mode will result in useful information.*

Whatever design is used, it is likely that conditions encountered in a scale-up effort will be less well suited for research than conditions encountered in efficacy and even effectiveness trials. Before initiating rigorous scale-up research, it is desirable to conduct an evaluability assessment to evaluate the likelihood that the evaluation of the intervention in scale-up mode will result in useful information.

Finally, the effectiveness section discussed the importance of embedding research to inform scale-up efforts into ongoing effectiveness trials. It goes without saying that research on outcomes of scale-up efforts can likewise embed research to answer important questions that will inform subsequent scale-up efforts.

The ideas presented in this section represent a major change from the standards for broad dissemination articulated a decade ago. These changes reflect the realization that the marketing of a research product can no longer be viewed as the end point of the preventive intervention research cycle. Instead, we concur with the view of Spoth et al. (2013) of the process of developing and testing preventive intervention as ongoing and cyclical. Information from earlier stages informs decisions about scaling up. What is learned from studies at later stages in the process is fed back to inform refinements of the interventions, which are then tested in accordance with the standards for efficacy and effectiveness trials articulated earlier.

## Conclusion

Establishing standards for Prevention Science is challenging. The field is evolving rapidly and it lacks consensus on several key issues. During the development of these revised standards, we debated several points that have great relevance for the future of the field but about which prevention scientists disagree. One of these is whether or not the field is well served by continuing to embrace the traditional preventive intervention research cycle articulated 20 years ago (Mrazek and Haggerty 1994). Another is the extent to which efforts to disseminate preventive interventions should be constrained by the rigorous standards of evidence articulated in this document.

While most prevention scientists recognize that attention to the translation of EBIs into population-level use has increased, some believe that this implies that the earlier stages of research (especially efficacy testing) are often not necessary. According to this perspective, the field should focus mainly on large-scale interventions and research on methods for effective scaling up of available practices. Proponents believe that the preventive intervention research cycle encourages research on smaller scale strategies that are amenable to testing through RCTs and discourages research on more scalable interventions. They further suggest that scaling up most developer-based individual-level preventive interventions that have been tested in RCTs is close to impossible and that research on such interventions should be discontinued in favor of research on policies and practices that are either already delivered at scale or that can feasibly be taken to scale.

A closely related issue pertains to the level of scientific rigor implied in the standards and, in particular, the requirement that EBIs meet all of the standards for efficacy and effectiveness before being scaled up. Some question our ability to meet increasing demand for prevention policies and services if we must limit the menu of available EBIs on the basis of rigorous evidence suggesting that these policies or practices have a reasonable probability of achieving a desirable outcome. Some believe that, although each of the standards articulated in this document is reasonable, the sum total of the standards place most potentially effective interventions outside the boundaries of what will be recognized as worthy of scale-up. Also, they point out that interventions evolve quickly once they are disseminated. Even if research on the initial EBI satisfied all of the standards, what should be done as it evolves?

Proponents of this perspective believe that we can afford to accept some error in decisions about which interventions to scale up. Legislators have to legislate, agencies have to create policies, and communities will try interventions with or without the input of prevention scientists. We have the ethical obligation to bring our best, even imperfect, evidence to bear on these decisions. Some believe that little harm will be done if we scale up EBIs that turn out not to be as effective as implied in rigorous research. In short, this perspective holds that we risk irrelevance and further separation from practice if we cannot meet the demand for more preventive services and that we cannot afford to limit our menu to the small fraction of possible interventions that have been rigorously tested.

Another point of view (and the one that is reflected in the revised standards) is that there is a critical need to develop and test new interventions that can become the building blocks of larger, effective prevention systems. Some of our “effective” interventions require further research and development to boost their small to negligible effects. Others require updated research to demonstrate continued effectiveness. Also, as the population changes and new behaviors emerge, additional research is needed to design new or modify existing preventive interventions to address current needs (e.g., cyberbullying). Thus, continued research to develop and modify preventive interventions is needed, and flexible use of the preventive intervention research cycle as recommended in these standards will enhance the quality of this research.

Research is needed to support both large-scale policy and legislative interventions and interventions targeting individuals. Despite the potential of larger scale interventions to reach large proportions of the population, history shows that many policy or legislative changes fail to change behavior. Demand for interventions aimed at providing effective services to individuals and families will always exist, and high-quality research will improve the menu of such services that can be made available. The goal in promoting these interventions should be that EBIs aimed at improving child care, social competency instruction, family services, etc. replace less effective practices currently in place in schools, clinics, mental health, and other youth-serving organizations. According to this perspective, such broad changes to the nature of services available to the population are as likely to bring about meaningful change as broad policy and legislative changes. The need for continued research at all of these levels remains high.

In response to those who believe that rigorous efficacy trials are appropriate only for small, individual-level interventions, we suggest that the challenges to using the research designs recommended in this document are often exaggerated. As discussed earlier in the document, rigorous methods for drawing causal inferences about the effects of interventions involving large aggregates exist, and the research literature now contains many examples of the use of these designs to study population-level interventions such as rural road paving, water quality improvement, poverty reduction, and teacher incentives.

Many of the concerns about the trade-offs between meeting demand for prevention and holding to rigorous standards of evidence might be resolved by recognizing that demand can be met in a variety of ways that do not involve making claims about the effectiveness of specific EBIs. This document establishes standards for the evidence that should be present to support such claims. These standards do not pertain to basic research in prevention or to a wide variety of prevention-related activities such as advocacy and communication about the nature of problems, precursors to dysfunction or well-being, and so on. A sizable improvement in many aspects of well-being may result simply from the dissemination of knowledge about conditions that are needed to ensure that children and adolescents develop successfully, even in the absence of specific EBIs. Agencies and legislators should look to prevention scientists for such evidence, and prevention scientists should assist. These standards seek mainly to constrain scientifically unsupported claims about the effectiveness of specific EBIs. The underlying assumption in providing these standards is that investing in high-quality research will produce higher quality evidence on which to base sound policies and practices. More informed decisions will enhance well-being more in the long run than will best guesses based on existing evidence.

This document updates SPR’s standards of evidence to reflect important changes that have occurred over the past decade that have implications for the design, implementation, and reporting of Prevention Science intervention trials. As noted in the “Introduction,” we chose to orient toward the future, envisioning changes to the status quo that could strengthen the impact of Prevention Science to improve the public health and well-being. By endorsing the standards contained in this document, SPR embraces a vision for the future that encourages the application of science to improve practices. This vision is consistent with Campbell’s (1968) ideal of the “experimenting society” in which actors remain committed to reality-testing and are self-critical and honest. They continually seek opportunities to test beliefs about the world against reality and remain open to what the data say. This experimental approach to improving the world implies an ongoing commitment to self-study consistent with this new generation of SPR standards.

## Footnotes

1 We are aware of the challenges related to identifying core components of an EBI and the fact that the rigorous research necessary to adequately test differential effects of different components of an EBI is rare in Prevention Science (Elliott and Mihalic 2004). We suggest that the identification of core components is provisional and based on the developers’ theory of the intervention, but at the same time encourage an increase in empirical testing of these components. See Efficacy *Standard 4*.

2 See effectiveness standard 6.a for discussion of meaning of “public health impact.”

3 This is not true of a similar design often used to study smaller scale interventions, primarily in behavioral analysis, the ABA or ABAB design. This design is similar to the time series design used for larger units except that the timing of the intervention is controlled by the researcher and therefore not confounded with other events.

4 They calculate the rate of erroneous published positive findings in the field of psychology as follows: Assume the null is true 90 % of the time. Using alpha = .05 and power = .8, type I error will occur in 4.5 % of studies (90 % *.05) and correct rejections of the null will occur in 8 % of studies (10 % *.8). Therefore, the proportion of published positive findings that are erroneous is 36 % (4.5 %/(4.5 % + 8 %)). The calculation is highly dependent upon the assumption about the percentage of tests conducted for which the null hypothesis is true. The actual rate of correct nulls in Prevention Science studies is not known.

5 Establishing generalizability across time (e.g., do observed effects of Head Start programs tested in the 1980s generalize to Head Start programs operating decades later?) and settings (e.g., do effects observed for Life Skills Training observed in public schools generalize to alternative schools?) may also be important. If the statement of “for whom” and “under what conditions” the intervention is expected to be effective (see Efficacy *Standard 2.c.*) includes specific times and settings, generalizability across these dimensions should also be tested.

6 Among the interventions for which at least one study conducted by an independent evaluator has failed to replicate findings reported by the developer are: Alert (Ringwalt et al. 2010); Multi-Systemic Therapy (Centre for Children and Families in the Justice System 2006; Löfholm et al. 2009); PATHS (Social and Character Development Research Consortium 2010); Quantum Opportunities (Schirm et al. 2006); Reconnecting Youth (Hallfors et al. 2006); Strengthening Families Program (Gottfredson et al. 2006; Gutman et al. 2004); Toward no Drug Abuse (Rohrbach et al. 2010); and Triple P (Malti et al. 2011). Note that for some of these interventions, independent evaluations have also found positive effects.

7 These practices include, “(a) leveraging chance by running many low-powered studies, rather than a few high-powered ones; (b) uncritically dismissing “failed” studies as pilot tests or because of methodological flaws but uncritically accepting “successful” studies as methodologically sound; (c) selectively reporting studies with positive results and not studies with negative results or selectively reporting “clean” results; (d) stopping data collection as soon as a reliable effect is obtained; (e) continuing data collection until a reliable effect is obtained; (f) including multiple independent or dependent variables and reporting the subset that “worked;” (g) maintaining flexibility in design and analytic models, including the attempt of a variety of data exclusion or transformation methods, and reporting a subset; (h) reporting a discovery as if it had been the result of a confirmatory test, and; (i) once a reliable effect is obtained, not doing a direct replication” (Nosek et al. 2012, p. 618).
